# Predicting microRNA targeting efficacy in Drosophila

**DOI:** 10.1186/s13059-018-1504-3

**Published:** 2018-10-04

**Authors:** Vikram Agarwal, Alexander O. Subtelny, Prathapan Thiru, Igor Ulitsky, David P. Bartel

**Affiliations:** 1 Whitehead Institute for Biomedical Research and Howard Hughes Medical Institute, 9 Cambridge Center, Cambridge, MA 02142 USA; 2 Department of Biology, Massachusetts Institute of Technology, Cambridge, MA 02139 USA; 3 Computational and Systems Biology Program, Massachusetts Institute of Technology, Cambridge, MA 02139 USA; 4 Present address: Department of Genome Sciences, University of Washington, Seattle, WA 98195 USA; 5 Department of Biological Regulation, Weizmann Institute of Science, Rehovot 76100, Israel; 6 Harvard-MIT Division of Health Sciences and Technology, Cambridge, MA 02139 USA

**Keywords:** Non-coding RNAs, miRNA target prediction, Post-transcriptional gene regulation

## Abstract

**Background:**

MicroRNAs (miRNAs) are short regulatory RNAs that derive from hairpin precursors. Important for understanding the functional roles of miRNAs is the ability to predict the messenger RNA (mRNA) targets most responsive to each miRNA. Progress towards developing quantitative models of miRNA targeting in Drosophila and other invertebrate species has lagged behind that of mammals due to the paucity of datasets measuring the effects of miRNAs on mRNA levels.

**Results:**

We acquired datasets suitable for the quantitative study of miRNA targeting in Drosophila*.* Analyses of these data expanded the types of regulatory sites known to be effective in flies, expanded the mRNA regions with detectable targeting to include 5′ untranslated regions, and identified features of site context that correlate with targeting efficacy in fly cells. Updated evolutionary analyses evaluated the probability of conserved targeting for each predicted site and indicated that more than a third of the Drosophila genes are preferentially conserved targets of miRNAs. Based on these results, a quantitative model was developed to predict targeting efficacy in insects. This model performed better than existing models, and it drives the most recent version, v7, of TargetScanFly.

**Conclusions:**

Our evolutionary and functional analyses expand the known scope of miRNA targeting in flies and other insects. The existence of a quantitative model that has been developed and trained using Drosophila data will provide a valuable resource for placing miRNAs into gene regulatory networks of this important experimental organism.

**Electronic supplementary material:**

The online version of this article (10.1186/s13059-018-1504-3) contains supplementary material, which is available to authorized users.

## Background

MicroRNAs (miRNAs) are ~ 22-nt regulatory RNAs that originate from hairpin precursors [1]. In Drosophila, they associate primarily with the Argonaute1 (dmAgo1) protein to form a silencing complex [2, 3] within which the miRNA functions as a sequence-specific guide that recognizes target messenger RNAs (mRNAs) through pairing to complementary sites primarily within the 3′ untranslated regions (3′ UTRs) [4–6].

The miRNA pathway found in flies is ancestral to animals [7], with dozens of miRNA genes conserved broadly in bilaterian species [8–11]. Small-RNA sequencing has identified hundreds of miRNAs that are encoded in fly genomes [8, 11–15], which in aggregate appear to target thousands of mRNAs [6, 8, 14, 16–20]. Studies of miRNAs in *Drosophila melanogaster* have helped define biological roles of miRNAs, components of the miRNA processing pathway, and evolutionarily conserved mechanisms of miRNA action [21–23].

Drosophila miRNAs are expressed in complex spatiotemporal patterns throughout development [24, 25] and play a wide diversity of roles. Examples include functions for bantam miRNA in the regulation of cell proliferation [26], miR-iab-4/iab-8 in body patterning [27–29] and behavior [30], miR-14 in insulin production and metabolism [31], miR-34 in aging and neurodegeneration [32], and miR-277 in branched-chain amino acid catabolism [33]. Indeed, a large-scale survey of miRNA knockouts in the flies reports abnormal knockout phenotypes for more than 80% of the miRNA genes tested [23].

Crucial for understanding the molecular basis of these phenotypes is the search for, and characterization of, miRNA targets. Analyses of reporter assays and site conservation indicate that the canonical site types identified in mammals, which include perfect Watson–Crick pairing to the miRNA seed (miRNA nucleotides 2–7) [34], also function in flies [6, 8, 16, 17, 19, 20, 35, 36]. However, knowledge of miRNA targeting in flies has lagged behind that of mammals, primarily due to the lack of high-throughput datasets examining the responses of mRNAs to the perturbation of miRNAs. In mammals, such datasets have been very useful for both measuring the relative efficacy of different site types and identifying additional features that influence site efficacy, such as those related to the context of the site within the mRNA, thereby enabling the development of quantitative models of site efficacy [5]. Although, as in mammals, much of miRNA targeting in flies is known to be seed-based, the relative importance of site types and context features might differ between mammals and flies, calling into question the utility for flies of quantitative models developed using mammalian data. For instance, fly 3′ UTRs are shorter and have a higher AU content than those of mammals, which would presumably affect the utility of context features such as distance from a 3′ UTR end or local AU content, which are known to be predictive of site efficacy in mammals [37]. Although some attempt to model the effect of target-site accessibility on miRNA-mediated repression has been applied to Drosophila as well as mammals [38], the relatively poor performance of this model when tested in mammalian systems suggests that in the fly it would have also benefited from the use of large datasets for training and validation [39].

Despite the lack of high-throughput repression data, many algorithms have been developed to predict and rank miRNA targets in Drosophila. Most, including European Molecular Biology Laboratory (EMBL) predictions [6, 40], EIMMo [41], MinoTar (also available as TargetScanFly ORF) [19], miRanda-MicroCosm [42], PicTar [16, 43], and TargetScanFly v6 [8], use a mix of pairing and evolutionary criteria, with pairing sometimes evaluated using predicted thermodynamic stability. Others, including PITA [38], RNA22 [44], and RNAhybrid [45], utilize purely thermodynamic information. Others, such as DIANA-microT-CDS [46], mirSVR [47], and TargetSpy [48], were trained on mammalian data using machine-learning strategies and then used to generate predictions for flies. Finally, ComiR integrates predictions from miRanda, PITA, TargetScanFly, and mirSVR, while being trained on the identities of RNAs that tend to co-purify with dmAgo1 [49].

As with most algorithms applied in mammals, some of those applied in flies predict many non-canonical target sites that have one or more mismatches or wobbles to the miRNA seed. However, others, including DIANA-microT-CDS, EIMMo, MinoTar, RNAhybrid, and TargetScanFly, require perfect seed pairing in an effort to enhance the specificity of detecting functional targets, although it is unclear to what degree this comes at the price of reduced sensitivity. Whereas most algorithms limit predictions to sites in 3′ UTRs, DIANA-microT-CDS and MinoTar also include predictions with sites in coding regions, which seem to have an even greater signal for preferential conservation in flies than they do in mammals [19, 34].

Here, we used RNA sequencing (RNA-seq) to monitor the effects of introducing specific miRNAs into Drosophila cells. Analyses of these data, together with updated analyses of site conservation in flies and other insects, provided new and quantitative insights into the types of target sites that function in flies, the scope of targeting in flies, and features of site context that influence site efficacy. With these insights, we generated a quantitative model that improves the rankings of target predictions for the fly miRNAs, available at TargetScanFly, v7 (http://www.targetscan.org). We also release an accompanying suite of computational tools to help others reproduce our figures and apply our analyses to future datasets (TargetScanTools; https://github.com/vagarwal87/TargetScanTools).

## Results and discussion

### Canonical miRNA target sites function primarily in Drosophila 3′ UTRs

To acquire datasets suitable for quantitative analysis of miRNA targeting in fly cells, we monitored the changes in mRNA levels after co-transfecting S2 cells with one of six different miRNA duplexes and a green fluorescent protein (GFP)-encoding plasmid. The six transfected miRNAs (miR-1, miR-4, miR-92a, miR-124, miR-263a, and miR-994) were chosen because they (or related miRNAs in the same seed family) were not endogenously expressed in S2 cells [8], and they had diverse starting-nucleotide identities, a range of GC content within their seeds, and a moderate-to-high range of predicted target-site abundances. After enriching for transfected, GFP-positive cells by fluorescence-activated cell sorting (FACS), mRNA-seq was performed, and mRNA fold changes were calculated for each miRNA transfection condition relative to a mock transfection, in which the GFP plasmid was transfected without any miRNA duplex (Additional file 1: Table S1). We then normalized the data to reduce batch effects (Additional file 2: Figure S1A–D), some of which were attributable to modest but statistically significant de-repression of the predicted targets of highly expressed endogenous miRNAs, such as bantam miRNA (Additional file 2: Figure S1E–G) [50, 51]. With this new dataset, we begin investigating the features of miRNA target sites that correlate with mRNA repression in Drosophila cells.

**Fig. 1:**
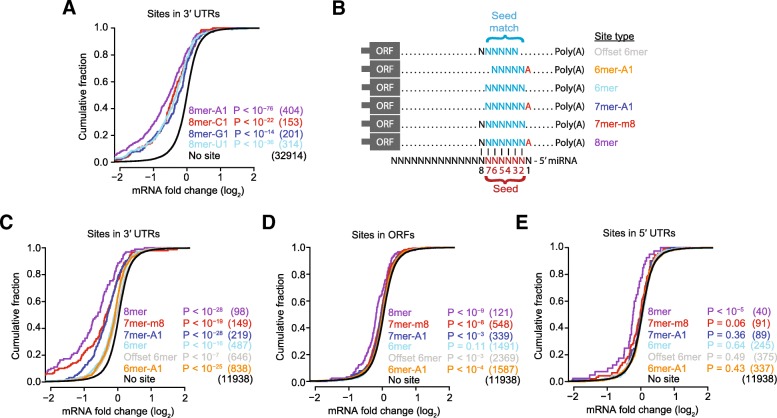
Drosophila miRNAs mediate mRNA repression through the targeting of canonical site types, preferentially in 3′ UTRs. **a** The increased efficacy in Drosophila of sites with an A across from miRNA position 1. Shown is the response of mRNAs to the transfection of a miRNA (either miR-1, miR-4, miR-92a, miR-124, miR-263a, or miR-994). Data were pooled across these six independent experiments. Plotted are cumulative distributions of mRNA fold changes observed upon miRNA transfection for mRNAs that contained a single site of the indicated type to the transfected miRNA. The site types compared are 8mers that perfectly match miRNA positions 2–7 and have the specified nucleotide (A, C, G, or U) across from position 1 of the miRNA. Also plotted for comparison is the cumulative distribution of mRNA fold changes for mRNAs that did not contain a canonical 7- or 8-nt site to the transfected RNA in their 3′ UTR (no site). Similarity of site-containing distributions to the no-site distribution was tested with the one-sided Kolmogorov–Smirnov (K–S) test (*P* values). Shown in parentheses are the numbers of mRNAs analyzed in each category. **b** The six canonical site types for which a signal for repression was detected after transfecting a miRNA into Drosophila cells. **c**–**e** The efficacy of the canonical site types observed in Drosophila 3′ UTRs (**c**), ORFs (**d**), and 5′ UTRs (**e**). These panels are as in **a**, but compare fold-change distributions for mRNAs possessing a single canonical site in the indicated region to those with no canonical sites in the entirety of the mRNA. See also Additional file 2: Figures S1 and S2

In mammals, the presence of an A opposite the first nucleotide of a miRNA is preferentially conserved and correlates with enhanced repression, regardless of the identity of the first nucleotide of the miRNA—observations explained by a pocket within human Argonaute2 (hsAGO2) that preferentially binds this A [34, 37, 39, 52]. In flies, an A at this position of the target site is also associated with enhanced conservation compared to otherwise identical sites missing this A [20], whereas in nematodes conservation and efficacy of a site with perfect pairing to miRNA nucleotides 2–8 followed by a U (8mer-U1 sites) resembles that of 8mer-A1 sites [20, 53, 54]. We therefore examined the influence of the nucleotide at target position 1 in flies, considering the data from all miRNA transfections pooled together. Of the mRNAs possessing a single match to miRNA nucleotides 2–8 in their 3′ UTR, those with an A opposite miRNA position 1 (i.e., those with the 8mer-A1 site) tended to be more repressed than those with each of the other three possibilities opposite miRNA position 1 (8mer-C1, 8mer-G1, and 8mer-U1, respectively), with the identity of the other three possibilities having little influence on repression (Fig. 1a). As expected based on the observation that the first position of the guide RNA is buried within Argonaute and unavailable for pairing [52, 55, 56], this observation generally held when considering each miRNA transfection independently, regardless of whether the identity of the first nucleotide of the miRNA was a U (Additional file 2: Figure S2). Thus Drosophila exhibits a preference for A at target position 1 resembling that of mammals, implying that this target nucleotide is recognized by a pocket within dmAgo1 resembling that of hsAGO2. With respect to nomenclature, these results further supported consideration of the 8mer-A1 site as the canonical 8mer site of Drosophila, as was done originally in mammals [34].

Analogous analyses of mRNA fold-change values in mammalian systems have demonstrated the function and relative efficacy of 8mer, 7mer-m8, 7mer-A1, 6mer, and offset 6mer sites [37, 57]. Accordingly, we examined the function of these site types in Drosophila, again pooling the data and focusing on mRNAs with a single site to the cognate miRNA. We also considered a sixth site type, the 6mer-A1 site, which has implied function in nematodes [20] and completes the set of all possible 8-, 7-, and 6-nt perfect matches to the 8-nt seed region, which we refer to as the canonical site types (Fig. 1b; note the distinction between the 6-nt seed and the 8-nt seed region). When located in the context of 3′ UTRs, each canonical site type was associated with repression, with the magnitude of repression following the hierarchy of 8mer > 7mer-m8 > 7mer-A1 > 6mer ~ offset 6mer ~ 6mer-A1 (Fig. 1c), as indicated from statistical testing of differences in fold-change distributions (Additional file 3: Table S2). This hierarchy resembled that of mammals, except that in mammals the efficacy of the different 6-nt sites is much more distinct, with 6mer > offset 6mer > 6mer-A1, and with the 6mer-A1 difficult to distinguish from background [37, 57].

We also examined the efficacy of canonical sites in mRNA regions outside of the 3′ UTR. Some repression was observed for mRNAs with a site in their open reading frame (ORF) (and no canonical site elsewhere in the mRNA), most convincingly for 8mer sites, although the efficacy of these sites was much less than that observed in 3′ UTRs (Fig. 1d). These observations are consistent with those in mammals [37, 58, 59]. In contrast to observations in mammals, however, repression was also observed for mRNAs with an 8mer site in their 5′ UTR (Fig. 1e). Taking these findings together, we conclude that miRNA targeting in flies resembles that of mammals, except that the efficacy of the three 6-nt canonical sites is more uniform in flies and repression of endogenous mRNAs is more readily detected in fly 5′ UTRs.

### Widespread conservation of canonical miRNA target sites in Drosophila UTRs

**Fig. 2:**
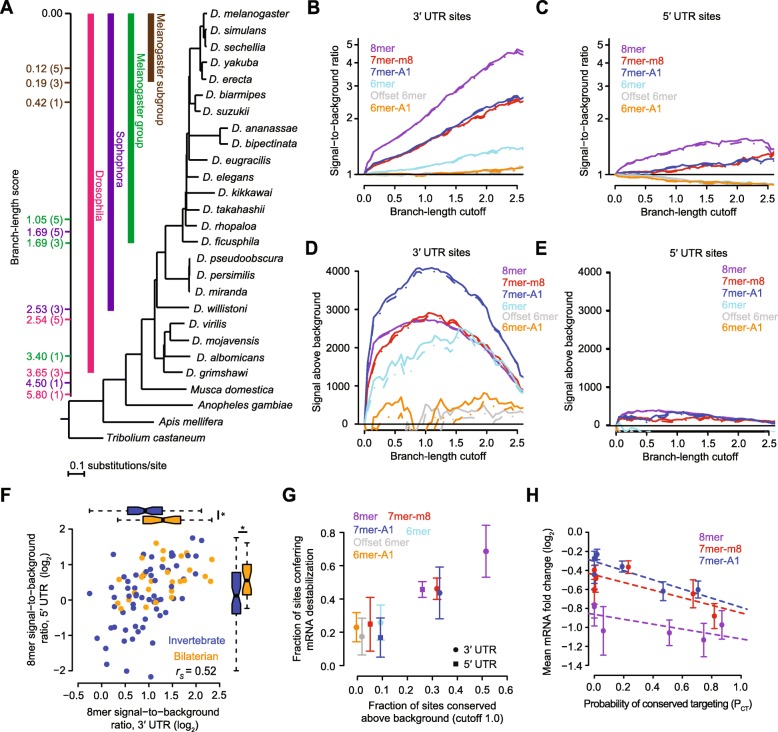
Evolutionary conservation of canonical sites in Drosophila 5′ UTRs and 3′ UTRs. **a** Phylogenetic tree of the 27 species used to examine miRNA site conservation. Outgroups of the genus *Drosophila* include *Musca domestica* (the housefly), *Anopheles gambiae* (the mosquito), *Apis mellifera* (the European honey bee), and *Tribolium castaneum* (the red flour beetle). *D. melanogaster* 3′ UTRs were assigned to one of five conservation bins based upon the median conservation of nucleotides across the entire 3′ UTR. The tree is drawn using the branch lengths and topology reported from genome-wide alignments in the UCSC Genome Browser. To the left of the tree, are *color-coded* branch-length scores corresponding to a site conserved among an entire subgroup of species indicated by a bar of the same color, showing scores for a site within a 3′ UTR in the lowest, middle, and highest conservation bins, labeled in parentheses as bins 1, 3, or 5, respectively. **b**, **c** Signal-to-background ratios for indicated site types at increasing branch-length cutoffs, computed for sites located in 3′ UTRs (**b**) or 5′ UTRs (**c**). *Broken lines* indicate 5% lower confidence limits (z-test). These panels were modeled after the one originally shown for the analysis of mammalian 3′ UTR sites [57]. **d**, **e** Signal above background for indicated site types at increasing branch-length cutoffs, computed for sites located in 3′ UTRs (**d**) or 5′ UTRs (**e**). *Broken lines* indicate 5% lower confidence limits (z-test). These panels were modeled after the one originally shown for the analysis of mammalian 3′ UTR sites [57]. **f** Signal-to-background ratios for the 8mer sites of 91 conserved miRNA seed families, calculated at near optimal sensitivity (a branch-length cutoff of 1.0), comparing the ratios observed for sites in 5′ UTRs to those for sites in 3′ UTRs (*r*_s_ Spearman correlation). Seed families conserved since the ancestor of bilaterian animals are distinguished from those that emerged more recently (*orange* and *blue*, respectively). *Boxplots* on the sides show the distributions of ratios for these two sets of families, with statistical significance for differences in these distributions evaluated using the one-sided Wilcoxon rank-sum test (*P < 0.01). See also Additional file 4: Table S3. **g** Relationship between site conservation rate and repression efficacy. The fraction of sites conserved above background was calculated as ([Signal – Background]/Signal) at a branch-length cutoff of 1.0. The minimal fraction of sites conferring destabilization was determined from the cumulative distributions (e.g., those in Additional file 2: Figure S2), considering the maximal vertical displacement from the no-site distribution (error bars, standard deviation, *n* = 6 miRNAs). *Colors* and *shapes* represent the canonical site types and UTR location, respectively. This panel was modeled after the one originally shown for the analysis of mammalian 3′ UTR sites [57]. **h** Relationship between site efficacy and site *P*_CT_. mRNAs were selected to have either one 7mer-A1, one 7mer-m8, or one 8mer 3′ UTR site to the transfected miRNA and no other canonical 3′ UTR site. mRNAs with sites of each type were grouped into six equal bins based on the site *P*_CT_. For each bin, mean mRNA fold change in the transfection data (error bars, standard error) is plotted with respect to the mean *P*_CT_, with the *dashed lines* showing the least-squares fit to the data. The slopes for each are negative and significantly different from zero (*P* value < 10^− 10^, linear regression using unbinned data)

A previous evolutionary analysis of mammalian miRNA target sites provided a framework for estimating the likelihood that predicted miRNA target sites are conserved across species, while controlling for factors such as differential species relatedness, differential background conservation in UTRs, and differential rates of dinucleotide substitutions [57]. Although this approach has also been applied to Drosophila genomes [20], we improved and extended it by (1) updating conserved miRNA family classifications and 3′ UTR annotations, (2) using an expanded evolutionary tree that incorporated additional insect species, (3) extending analyses to Drosophila 5′ UTRs, (4) using a modified evolutionary analysis pipeline [51], and (5) comparing our evolutionary results to our functional data. Towards this end, we compiled miRNA annotations from multiple studies [8, 10, 11, 15] and classified 91 miRNA families as broadly conserved among Drosophila species, 29 of which have been conserved since the last bilaterian ancestor (Additional file 4: Table S3). We also extracted multiple sequence alignments corresponding to annotated *D. melanogaster* 5′ UTRs and 3′ UTRs, assigning each UTR to one of five bins based on its background UTR conservation rates [20]. For each bin, we computed phylogenetic trees with a fixed species tree topology that encompassed 27 insect species, allowing for variable branch lengths to capture slower or faster substitution rates among the UTRs of the bin (Fig. 2a). These trees were then used to assign a branch-length score (BLS) [17] to each motif occurrence in *D. melanogaster* UTRs, which quantified the extent of conservation of that occurrence while controlling for the background conservation rate of its overall UTR context [57]. For example, a motif occurrence detected among all Sophophora species in the 3′ UTR alignment would be assigned a BLS of 4.50, 2.53, or 1.69, depending upon whether the corresponding 3′ UTR in which it resided was in the first, third, or fifth conservation bin, respectively (Fig. 2a).

For each site type of each of the 91 broadly conserved miRNA families, we computed the “signal” as the number of times that site occurred in *D. melanogaster* UTRs and had a BLS that equaled or surpassed a particular value (i.e., the “branch-length cutoff”). In parallel, we also computed the “background” as the number of conserved occurrences expected by chance, based upon the mean fraction of conserved motif instances for 50 length-matched *k*-mer controls, each of which was predicted to have background conservation resembling that of the miRNA site, as estimated from aggregated dinucleotide conservation rates [57]. This allowed us to compute a signal-to-background ratio at each branch-length cutoff, which represented the estimated enrichment of preferentially conserved miRNA sites in fly UTRs (Fig. 2b and c). It also allowed us to compute the signal above background, which represented the estimated number of miRNA sites that have been preferentially conserved in fly UTRs (Fig. 2d and e).

As expected, the signal-to-background ratios increased as the evolutionary conservation criteria became more stringent, with 8mers in 3′ UTRs reaching a ratio of nearly five conserved sites for every one control site at the greater branch-length cutoffs (Fig. 2b). For each site type, the ratios were consistently greater in the 3′ UTRs than they were in 5′ UTRs (Fig. 2b and c). For example, in 5′ UTRs the signal-to-background ratio for 8mers did not surpass 1.6 (Fig. 2c). These results showed that sites are more likely to be conserved if they reside in 3′ UTRs, presumably because this is where they are also more effective (Fig. 1). Nonetheless, when comparing the signal-to-background ratios for different miRNA families, ratios in 5′ UTRs correlated with those in 3′ UTRs (Fig. 2f; Additional file 4: Table S3). The greatest ratios tended to be for the fly miRNA families that have been conserved since the ancestor of bilaterian animals (Fig. 2f), as might be expected for these ancient families that have had more time to acquire more roles in gene-regulatory networks.

Although the sequence-conservation signal-to-background hierarchy of 8mer > 7mer > 6mer observed in both 5′ and 3′ UTRs matched the hierarchy observed for efficacy, some differences were observed. Most notably, the conservation signal for the 6mer site was robustly above background, whereas those for the offset 6mer and 6mer-A1 sites were both indistinguishable from background (Fig. 2b), even though these three 6-nt sites had similar efficacies in our repression data (Fig. 1c). Conversely, the 5′ UTR 7mer-A1 site exhibited a detectable signal for conservation (Fig. 2b), even though it had no detectable efficacy in mediating repression (Fig. 1c).

For sites in both 3′ and 5′ UTRs, the signal above background peaked near a branch-length cutoff of 1.0 (Fig. 2d). At this and other branch-length cutoffs, the signal above background was far higher in the 3′ UTR than in the 5′ UTR (Fig. 2d and e), which can be attributed to both a higher fraction of the sites preferentially conserved in 3′ UTRs, as indicated by the higher signal-to-background ratio in 3′ UTRs, and more sites residing in 3′ UTRs, mostly a consequence of 3′ UTRs generally being longer than 5′ UTRs. Including site types whose lower 5% confidence intervals exceeded zero, our results provided an estimate of ~ 12,285 sites conserved above background in 3′ UTRs (2738 ± 31 8mer, 2837 ± 68 7mer-m8, 4062 ± 100 7mer-A1, 2128 ± 221 6mer sites, and 520 ± 244 offset 6mer sites, calculated at a branch-length cutoff of 1.0 and reported ±90% confidence interval) (Fig. 2d). When added to our estimate of ~ 840 sites conserved above background in 5′ UTRs (350 ± 18 8mer, 165 ± 46 7mer-m8 sites, and 325 ± 44 7mer-A1 sites) (Fig. 2e), the estimated number of preferentially conserved UTR sites in Drosophila UTRs totaled ~ 13,125. Simulations that considered all of the conserved instances of site types, and then accounted for those that were estimated to be conserved by chance in 5′ UTRs and 3′ UTRs, indicated that these 13,125 preferentially conserved sites reside within 5035 ± 83 (90% confidence interval) of the 13,550 unique mRNAs with annotated UTRs of Drosophila, implying that mRNAs from 37.2% ± 0.6% of the Drosophila genes are conserved targets of the broadly conserved miRNAs.

Additional comparison of the results from our analyses of site conservation and site efficacy revealed that, as observed for mammalian 3′ UTR sites [57], there was a striking correlation between the fraction of sites conserved above background for each site type and the corresponding fraction of sites mediating mRNA destabilization (Fig. 2g). Slightly deviating from this trend were 3′ UTR 6mer-A1 sites, which appeared to mediate some repression despite lacking a signal for conservation, and 5′UTR 7mer-A1 sites, which had a modest signal for conservation despite undetectable efficacy of repression (Fig. 2g).

To estimate the extent to which each instance of each of the three most effective sites has been preferentially conserved, we computed the probability of conserved targeting (*P*_CT_) score for each of the 8mer, 7mer-m8, and 7mer-A1 sites residing in *D. melanogaster* 3′ UTRs. *P*_CT_ scores, which range from 0 to 1, summarize the estimated probability that a given site has been evolutionarily conserved because of its pairing to the cognate miRNA, while controlling for other factors, such as its length, surrounding genomic context, and dinucleotide content [57]. These scores provide a valuable resource for biologists wanting to focus on conserved targeting interactions. They also can help predict targeting efficacy [51, 57]. Indeed, sites with greater *P*_CT_ scores tended to confer more repression (Fig. 2h), implying that as expected, conserved sites were more likely to reside within contexts that favored their efficacy.

### Features useful for predicting site efficacy in flies

**Fig. 3:**
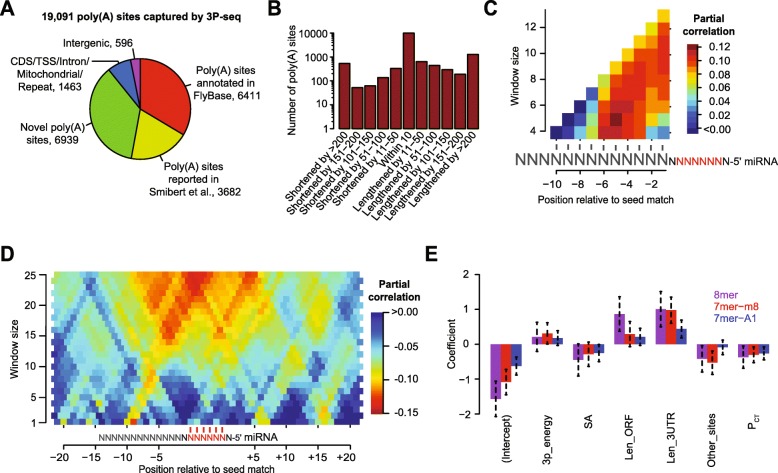
Refinement of 3′ UTR annotations in S2 cells and development of a regression model that predicts miRNA targeting efficacy in Drosophila. **a** Poly(A) sites detected in S2 cells by 3P-seq, classified with respect to their previous annotation status. **b** Extension and contraction of longest 3′ UTR isoforms relative to the FlyBase annotations. For each gene with a poly(A) site detected using 3P-seq, the difference between the longest 3′ UTR isoform annotated using 3P-seq was compared to longest 3′ UTR isoform annotated at FlyBase. These differences were then binned as indicated, and the number of sites assigned to each bin is plotted. **c** Optimization of scoring of predicted 3′ supplementary pairing in flies. Predicted thermodynamic energy scores were computed for the pairing between a 9-nt region upstream of canonical 7–8-nt 3′UTR sites and a variable-length region of the miRNA with the indicated size (window size) that began at the indicated position of the miRNA. The *heatmap* displays the partial correlations between these scores and the repression associated with the corresponding sites, determined while controlling for site type. **d** Optimization of the scoring of predicted structural accessibility in flies. Predicted RNA structural accessibility scores were computed as the average pairing probabilities for variable-length (window size) regions that centered at the indicated mRNA position, shown with respect to the seed match of each canonical 7–8-nt 3′ UTR site. The *heatmap* displays the partial correlations between these values and the repression associated with the corresponding sites, determined while controlling for site type. **e** The contributions of site type and each of the six features of the context model. For each site type, the coefficients for the multiple linear regression are plotted for each feature. Because features were each scored on a similar scale, the relative contribution of each feature in discriminating between more or less effective sites was roughly proportional to the absolute value of its coefficient. Also plotted are the intercepts, which roughly indicate the discriminatory power of site type. *Bars* indicate the 95% confidence intervals of each coefficient. See also Additional file 2: Table S4, Table S5, and Figure S3A

Before beginning to explore the features of site context associated with site efficacy, we improved the 3′ UTR annotations in S2 cells, the cell line in which we had acquired our functional data. We reasoned that more accurate annotation of these UTRs would allow us to reduce the impact of false-positive sites while appropriately weighting sites by the frequency of their inclusion within 3′ UTR isoforms [51, 60]. Knowledge of abundant alternative 3′ UTR isoforms for the mRNAs of a gene would also provide a more informed assessment of 3′ UTR-related features, such as 3′ UTR length and distance from the closest 3′ UTR end. Accordingly, we identified and quantified the 3′ UTR isoforms of S2 cells using poly(A)-position profiling by sequencing (3P-seq) [20]. Although the majority of the 3P-seq-supported poly(A) sites corresponded to either 3′ UTR isoforms that had been previously annotated by FlyBase or a large-scale study that annotated additional poly(A) sites [61], nearly 47% of the 3P-seq-supported poly(A) sites did not correspond to existing annotations, and most of these novel sites could be linked to a nearby gene with the support of RNA-seq evidence (Fig. 3a). In cases in which the longest 3′ UTR isoform for a gene annotated using 3P-seq differed from that annotated in FlyBase, it was more often longer, although for nearly 1000 genes the 3P-seq results implicated the dominant use of a shorter 3′ UTR isoform in S2 cells (Fig. 3b). Using this information, we compiled a set of 3826 mRNAs that passed our expression threshold in S2 cells and for which ≥ 90% of the 3P-seq tags corresponded to a single dominant 3′ UTR isoform in these cells, and we used this set to investigate features of site context associated with site efficacy.

With this set of mRNAs and repression values in hand, we examined two of the more complex features of site context, confirming their effects in Drosophila cells and developing scoring schemes that best correlated with their influence in these cells. The first of these two features was 3′ supplementary pairing, i.e., pairing to the target by miRNA nucleotides outside of the seed region. The strength of this pairing was evaluated as the predicted thermodynamic energy of pairing between the 3′ region of the miRNA and a corresponding mRNA region upstream of the seed match. This predicted energy of pairing was evaluated for mRNAs that possessed a single 7–8-nt 3′ UTR site for the transfected miRNA and then compared to the repression observed for the mRNAs upon miRNA transfection by computing a partial correlation between 3′ supplementary pairing energies and mRNA changes, controlling for site type.

In mammalian cells, 3′ supplementary pairing is most influential when centered on nucleotides 13–17 [37], but in flies the pairing possibilities most consequential for repression had not been identified. To systematically examine these possibilities, we varied three parameters: (1) the start position of the miRNA region considered, examining all start possibilities from positions 9 to 19, (2) the length of the miRNA region considered, examining lengths from 4 to 13 nt, and (3) the length of the target region upstream of the seed match, examining lengths from 4 to 20 nt. A grid search over all parameter combinations revealed that the predicted energy of 3′ supplementary pairing energy was optimally predictive of repression efficacy when it was calculated for the pairing that can occur between miRNA nucleotides 13–17 and a 9-nt region upstream of the seed match (Fig. 3c).

The second feature we investigated was the influence of 3′ UTR structure on target-site accessibility. This feature has been evaluated previously using two approaches, either evaluating nucleotide composition near the site, reasoning that sites residing in high local AU content would be more accessible [37], or attempting to predict site accessibility using various RNA-folding algorithms [38, 51, 62–65]. With respect to the second approach, a method originally developed to predict small interfering RNA (siRNA) target-site accessibility [62] appears to be one of the more effective methods for predicting miRNA target-site accessibility in mammals [51]. This method folds the 80-nt region centered on the seed match and then reports a structural accessibility (SA) score calculated as the mean unpaired probabilities for a smaller window in the vicinity of the seed match [51, 62]. To determine the optimal location and width of this window for scoring SA in flies, we again computed partial correlations, this time between mean pairing probabilities and mRNA changes, varying two parameters: (1) the position of the center of the window within the target mRNA, examining each position within 20 nt of the seed match, and (2) the size of this window, considering sizes of 1 to 25 nt. A grid search over all parameter combinations indicated that a 25-nt window centered on the nucleotide that pairs to miRNA position 7 was optimal for calculating SA in flies (Fig. 3d). Although the optimal window size fell at the edge of the range, larger windows were not considered because they were more prone to extend beyond 3′ UTR boundaries, which reduced the sample size.

### A quantitative model for predicting site efficacy in flies

To identify and evaluate additional features associated with site efficacy in flies and generate a resource for placing fly miRNAs into gene regulatory networks, we developed a quantitative model of miRNA targeting efficacy for flies, which resembled our models developed for mammals [37, 51, 66]. The smaller scope of our fly dataset imposed some limitations on the features we could examine in flies as well as the strategy used to train the model. In particular, the number of training examples was an order of magnitude lower in the fly dataset relative to the human dataset. This was due to (1) fewer small-RNA transfection datasets in S2 cells compared to those available in HeLa cells, (2) a smaller number of genes expressed in S2 cells compared to those expressed in HeLa cells, and (3) shorter 3′ UTRs in flies, which further decreased the number of 3′ UTRs with a site for a miRNA of interest. Thus, we did not consider features related to the identity of the miRNA seed, such as estimated target-site abundance within the transcriptome, predicted seed-pairing stability, and nucleotide identity at the miRNA or target position 8, which are each informative for predicting targeting efficacy in human cells [51, 66]. Moreover, rather than considering features for each site type independently, we trained a single, unified regression model that considered the site type itself as a potential feature of targeting. In addition to site type, seven other features of the sites and their surrounding context and nine features of the target mRNAs were considered as potentially informative of targeting efficacy, either because they had been previously shown to correlate with targeting efficacy in flies or mammals, or because they were related to features shown to correlate with efficacy (Table 1).

**Table 1:** The 17 features considered in the models, highlighting the seven robustly selected through stepwise regression (in boldface text). The feature description does not include the scaling performed (Additional file 2: Table S4) to generate more comparable regression coefficients

Feature	Abbreviation	Description	Frequency chosen
Site			
**Site type**	**site type**	**Type of site (8mer, 7mer-m8, or 7mer-A1)** [37]	**100%**
Site position 9	site9	Identity of nucleotide at position 9 of the site	2%
Site position 10	site10	Identity of nucleotide at position 10 of the site	0%
Local AU content	local_AU	AU content within 30 nucleotides of the site [37]	51%
3′ supplementary pairing	3P_score	Supplementary pairing at the miRNA 3′ end [37]	4%
**Energy of 3′ supplementary pairing**	**3P_energy**	**Thermodynamic energy of supplementary pairing at the miRNA 3′ end (ΔG duplex – ΔG seed duplex) (**Fig. 3c**)**	**94%**
**Predicted structural accessibility**	**SA**	**log**_**10**_**(Probability that a 25-nt segment centered on the match to miRNA position 7 is unpaired) (**Fig. 3d**)**	**92%**
**Probability of conserved targeting**^a^	*P*_**CT**_	**Probability of site conservation, controlling for dinucleotide evolution and site context** [57]	**100%**
mRNA			
5′ UTR length	len_5UTR	log_10_(Length of the 5′ UTR)	30%
**ORF length**	**len_ORF**	**log**_**10**_**(Length of the ORF)** [51]	**100%**
**3′ UTR length**	**len_3UTR**	**log**_**10**_**(Length of the 3′ UTR)** [91]	**100%**
5′ UTR AU content	AU_5UTR	Fractional AU content in the 5′ UTR	17%
ORF AU content	AU_ORF	Fractional AU content in the ORF	37%
3′ UTR AU content	AU_3UTR	Fractional AU content in the 3′ UTR	56%
Distance from stop codon	dist_stop	log_10_(Distance of site from stop codon)	4%
Minimum distance	min_dist	log_10_(Minimum distance of site from stop codon or poly(A) cleavage site) [37, 41, 92]	55%
**Weak canonical sites in mRNA**	**other_sites**	**Number of 8mer sites in the 5′ UTR and ORF and offset-6mer, 6mer-A1, and 6mer sites in the 3′ UTR** [51]	**100%**

^a^Only relevant for deeply conserved miRNA families

Starting with these features, we trained models of targeting efficacy using a variety of machine-learning algorithms. To evaluate each algorithm, we partitioned our dataset into 1000 bootstrapped samples to estimate the held-out prediction performance. Each sample included 70% of the mRNAs with a single 7–8-nt 3′ UTR site from each miRNA transfection experiment (randomly selected without replacement); we reserved the remaining 30% for testing. Among the different algorithms, a stepwise regression strategy that maximized the Akaike information criterion (AIC) led to the best empirical performance (Additional file 2: Figure S3A). This stepwise regression strategy was the same algorithm that we had recently used to build a model of mammalian miRNA targeting efficacy [51]. Relative to a model that considered only site type (the “site only” model), the stepwise regression model that considered features of site context was twofold to threefold improved in predicting the mRNA fold-change measurements (median *r*^*2*^ of 0.08 and 0.19, respectively; *P* < 0.001, paired Wilcoxon signed-rank test; Additional file 2: Figure S3A).

At first glance, an *r*^*2*^ of only 0.19 for the best algorithm might seem to be a concern, as it implies that the method accounts for only 19% of the variability observed in our datasets. However, no model of miRNA targeting can explain variability arising from either experimental noise or the secondary effects of repressing the primary targets, which together contribute a large fraction of the variability observed in miRNA transfection datasets. Indeed, our analysis of the changes observed for predicted targets of one miRNA when another miRNA was transfected indicated that experimental noise and secondary effects together accounted for nearly half of the variability observed in our datasets, implying that a perfect model of direct targeting could explain at most 52% of the variability (Additional file 2: Figure S3B). Thus, the *r*^*2*^ of 0.19, which resembled that obtained in mammalian analyses [51], implied that the model explained ~ 37% of the variability attributable to direct targeting.

The features most informative for the stepwise regression model were presumably those with the greatest impact on site efficacy in flies. To identify these key features, we quantified the percentage of bootstrapped samples in which each feature was chosen (Table 1). Seven of the 17 features were selected in ≥ 90% of the bootstrap samples (Table 1), and multiple linear regression models trained with only these seven features performed at least as well as those that considered all 17 features (median *r*^*2*^ of 0.20; Additional file 2: Figure S3A). Aside from site type, which has long been considered in TargetScanFly [8], these robustly selected features included three features of the site: energy of 3′ supplementary pairing (3P_energy), SA, and evolutionary conservation (*P*_CT_); and three features of the mRNA: ORF length (len_ORF), 3′ UTR length (len_3UTR), and the number of weak sites within the mRNA (other_sites) (Table 1). Notably, all of these features were previously selected when modeling site efficacy in mammals [51], with the nuance that in flies 3P_energy outperformed 3P_score, another method of evaluating 3′ supplementary pairing which had been optimized on mammalian data [37]. However, two features strongly associated with site efficacy in mammals were not consistently selected in the fly analysis. These included AU composition in the vicinity of the target site (local_AU) and the minimum distance of a site from 3′ UTR boundaries (min_dist) [37]. Perhaps these features did not strongly discriminate effective targets from ineffective ones in flies because compared to mammalian 3′ UTRs, fly 3′ UTRs are constitutively more AU-rich and much shorter. (The median 3′ UTR length is 661 nt and 202 nt for human and fly, respectively, considering the longest UTR annotation per gene after removing genes with the longest UTR annotations ≤2 nt.)

Using the seven consistently selected features and the entire dataset of 3′ UTRs containing single 7mer-A1, 7mer-m8, or 8mer sites, we trained independent multiple linear regression models for each of these three canonical sites. These three models were then combined to generate a model for fly miRNA targeting, which we call the “context model” because it resembled our context models developed for mammalian miRNA targeting in that it modeled site context in addition to site type. The sign of each coefficient revealed the relationship of each feature to repression (Fig. 3e). For example, mRNAs with longer ORFs or longer 3′ UTRs, and sites with weaker 3′ supplementary pairing energy were more refractory to repression (as indicated by a positive coefficient), whereas target sites that were more structurally accessible or more conserved, and mRNAs with other weak sites were more prone to repression (as indicated by a negative coefficient). Normalizing the scores of each feature to a similar scale enabled assessment of the relative contribution of each feature to the context model (Fig. 3e). As expected, site type was also a major predictor of repression in the model, as indicated by the large magnitude of the intercept term (Fig. 3e). The signs and relative magnitudes of the features largely paralleled those found in the mammals [51], indicating that the influence of these features might reflect evolutionarily conserved aspects of miRNA targeting in bilaterian species. One difference was that *P*_CT_ scores contributed relatively more to the fly context model than they do to the analogous mammalian model [51], implying that the detection and scoring of the molecular features of target efficacy have more room for improvement in flies, presumably because less data were available in flies for feature identification and evaluation.

### Comparison to the performance of previous methods

We next compared the performance of the fly context model to that of previously reported methods, measuring how successfully each method predicted and ranked the mRNAs that respond to the gain or loss of a miRNA in Drosophila. For training, our context model had considered only mRNAs that had a single 7–8-nt site to the cognate miRNA within their 3′ UTR, but for testing it needed to be extended to mRNAs that had multiple sites to the same miRNA within their 3′ UTRs. Accordingly, for each predicted target, we generated a total context score, calculated as the sum of the context scores of the sites to the cognate miRNA [37], and used these total context scores to rank all of the predicted targets for each miRNA. The response of the top-ranked targets was then compared to that of 14 previously reported methods, chosen because predictions for Drosophila targets were available online, as was information needed to rank the predictions. Having already generated the *P*_CT_ scores of the Drosophila sites, we also combined the scores of multiple 7–8-nt canonical sites when present within the same 3′ UTRs to generate Aggregate *P*_CT_ scores, which were also used to rank predictions based solely on the probability that they were preferentially conserved targets of the miRNA [57].

We took precautions to perform a fair comparison of the algorithms. First, for each algorithm, we considered only predicted targets that corresponded to mRNAs expressed above the quantification threshold in the relevant test-set sample lacking the miRNA. Second, we avoided testing the context model on the same transfection data upon which it was trained. More specifically, we implemented a cross-validation strategy when testing the results of the context model using the transfection datasets, sequentially holding out each dataset and retraining the coefficients for the features in our context model using the five remaining transfection datasets before generating predictions for the held-out dataset. Further reducing the concern of overfitting was the observation that most top-ranked targets contained two or more canonical 3′ UTR sites and thus were not used during the development and training of our model. Third, for all testing of the context model, we used coefficients retrained on publicly available FlyBase 3′ UTR annotations, reasoning that training on improved 3′ UTR annotations derived from our 3P-seq data would have imparted an advantage to our model.

Another key consideration for the fair comparison of prediction performance is the choice of the approach used to evaluate performance. The use of standard methods for evaluating a binary classifier, such as a receiver operating characteristic (ROC) curve, is not appropriate for several reasons. First, for miRNA target predictions, there is no suitable set of known true positives or true negatives, because databases of validated targets miss many of the actual targets and are strongly biased in favor of the prediction algorithms used to identify the target candidates that are then validated. In the absence of suitable sets of known positives and negatives, ROC analyses can be performed using the molecular effects of perturbing the miRNA, but this approach requires choosing a threshold to separate mRNAs that respond from those that do not. Choosing a stringent threshold misses many of the authentic targets, whereas choosing a less stringent threshold that has a chance of capturing most of the authentic targets brings in too many false positives. The problems with ROC curves compound when trying to compare the performance of different algorithms, some of which predict 100 times more targets than others. Picking a high-stringency cutoff does not do justice to the algorithms that provide many predictions with the goal of achieving greater prediction sensitivity, whereas picking a low-stringency cutoff is unfair to the algorithms that provide relatively few predictions in an effort to achieve greater prediction specificity. Moreover, the use a binary threshold obscures how accurately the algorithms rank their predicted targets. For these reasons, recasting the quantitative phenomenon of miRNA targeting as a binary classification problem is not appropriate, and fairly comparing prediction performance using ROC curves is not possible.

**Fig. 4:**
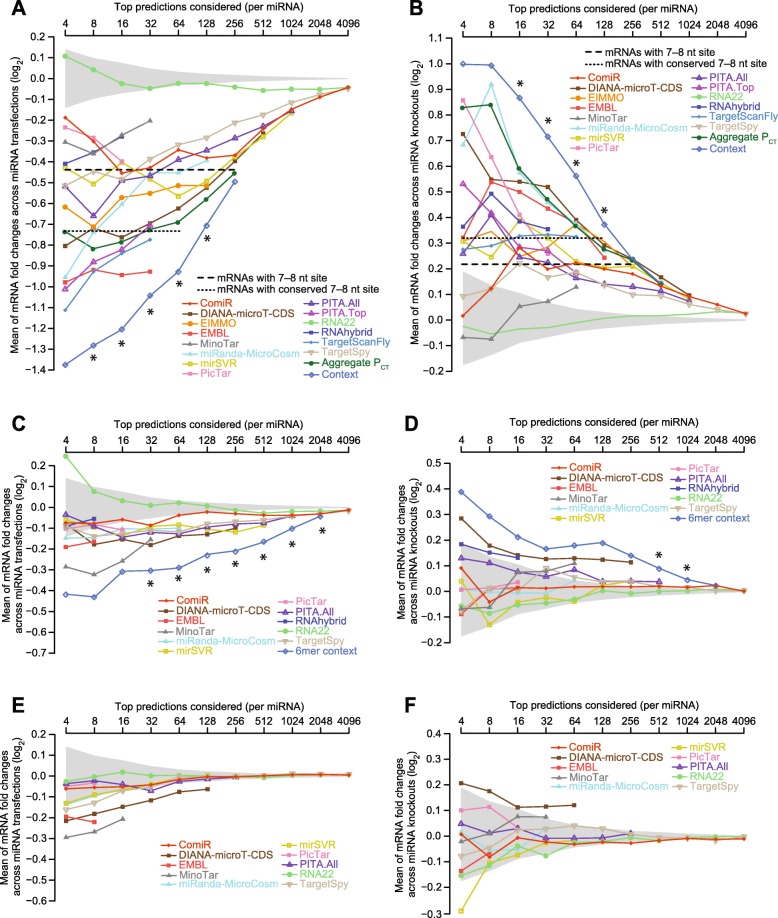
Performances of different target-prediction algorithms in flies. **a** The differential ability of algorithms to predict the mRNAs most responsive to miRNAs transfected into Drosophila cells. Shown for each algorithm in the *key* are mean mRNA fold changes observed for top-ranked predicted targets, evaluated over a sliding sensitivity threshold using the six miRNA transfection datasets. Some methods, such as PicTar, which generated relatively few predictions, could be evaluated at only a few thresholds, whereas others, such as RNA22 and TargetSpy, could be evaluated at many more. For each algorithm, predictions for each of the six miRNAs were ranked according to their scores, and the mean fold-change values were plotted at each sensitivity threshold. For example, at a threshold of 16, the 16 top predictions for each miRNA were identified (not considering predictions for mRNAs expressed too low to be accurately quantified). mRNA fold-change values for these predictions were collected from the cognate transfections, and the mean fold-change values were computed for each transfection for which the threshold did not exceed the number of reported predictions. The mean of the available mean values was then plotted. Also plotted are the mean of mean mRNA fold changes for all mRNAs with at least one cognate canonical 7–8-nt site in their 3′ UTR (*dashed line*), the mean of mean fold change for all mRNAs with at least one conserved cognate canonical 7–8-nt site in their 3′ UTR (*dotted line*) and the 95% confidence interval for the mean fold changes of randomly selected mRNAs, determined using 1000 resamplings (without replacement) at each cutoff (*shading*). Sites were considered conserved if their branch-length scores exceeded a cutoff with a signal:background ratio of 2:1 for the corresponding site type (cutoffs of 1.0, 1.6, and 1.6 for 8mer, 7mer-m8, and 7mer-A1 sites, respectively; Fig. 2b). Thresholds at which the distribution of fold changes for predicted targets of the context model was significantly greater than that of any other model are indicated (*, one-sided Wilcoxon rank-sum test, *P* value < 0.05). See also Additional file 2: Figure S4. **b** The differential ability of algorithms to predict the mRNAs most responsive to knocking out miRNAs in flies. Shown for each algorithm in the *key* are mean mRNA fold changes observed for top-ranked predicted targets, evaluated over a sliding sensitivity threshold using the three knockout datasets. Otherwise, this panel is as in **a**. **c** and **d** The differential ability of algorithms to predict targets that respond to the miRNA despite lacking a canonical 7–8-nt 3′ UTR site. These panels are as in **a** and **b**, except they plot results for only the predicted targets that lack a canonical 7–8-nt site in their 3′ UTR. Results for our context model and other algorithms that only predict targets with canonical 7–8-nt 3′ UTR sites are not shown. Instead, results are shown for a 6mer context model, which considers only the additive effects of 6mer, offset 6mer, and 6mer-A1 sites and their corresponding context features. **e** and **f** The difficulty of predicting mRNAs that respond to miRNA transfection or knockout despite lacking canonical 6–8-nt 3′ UTR sites. These panels are as in **c** and **d**, respectively, except they plot results for mRNAs with 3′ UTRs that lack a canonical 6–8-nt site

Recognizing these issues, a new approach has been developed for evaluating miRNA target-prediction performance [67], which we first implemented using our six datasets that each examined mRNA changes after transfecting a miRNA into S2 cells (Fig. 4a). For each algorithm and each transfected miRNA, we computed the mean mRNA fold change of the top-ranked targets of the transfected miRNA and then plotted the mean value for the six different miRNAs at various ranking thresholds, thereby summarizing repression efficacy of the top-ranked targets at each threshold. This approach of plotting mean repression over a range of ranking thresholds has several key features that make it suitable for fairly comparing target-prediction performance: (1) It is designed to test performance using global molecular measurements and thus does not require knowledge of true positives and true negatives, (2) it uses a sliding threshold and thus allows for simultaneous comparisons at all stringency cutoffs, (3) its sliding threshold is well suited for evaluating the ability of algorithms to rank predicted targets (given by the relationship between mean repression and stringency threshold).

When applying this analysis of performance, we found that all algorithms except RNA22 predicted repressed targets better than expected by chance (Fig. 4a). However, some, including ComiR, PicTar, MinoTar, RNAhybrid, TargetSpy, and mirSVR, performed similarly or worse than a naïve strategy of selecting all mRNAs that have at least one 7–8-nt canonical site in their 3′ UTR. Of the previously reported algorithms, TargetScanFly, EMBL, and PITA.Top performed the best. Nevertheless, our context model performed better than all previous methods, providing predictions that were the most responsive to transfection of the miRNA at each threshold tested (Fig. 4a).

Although our cross-validation strategy avoided testing our model on the same measurements as used for its training, some concerns regarding testing on the transfection data remained, because these data were used to optimize scoring of some features of our model. Moreover, transfection introduces high concentrations of miRNAs into cells in which they normally are not acting, raising the concern that a model developed and tested solely on transfection datasets might not accurately predict the response of miRNAs in their endogenous physiological contexts. Therefore, we searched for a test set that had not been used to develop any of the algorithms and that monitored the transcriptome response to endogenous miRNAs expressed at physiological levels. Instead of monitoring the new repression observed upon ectopic addition of a miRNA, such a test set would examine the de-repression observed upon loss of an endogenous miRNA. Surveying the Drosophila literature, we identified three miRNA knockout datasets with compelling signals for de-repression. Pooling these datasets, which monitored mRNA changes after deleting either miR-14 [31], miR-34 [32], or miR-277 [33], and carrying out the same type of analysis as we had done for the transfection datasets (but monitoring de-repression following loss of a miRNA instead of repression following introduction of a miRNA) revealed performances that generally resembled those observed with the transfection datasets (Fig. 4b). The relative performances of the previous methods shifted somewhat, with improvement observed for Aggregate *P*_CT_, miRanda-MicroCosm, and PicTar and worsening observed for MinoTar, TargetScanFly, and TargetSpy. Importantly, however, when testing on these consequences of endogenous miRNA targeting in flies, the context model again performed better than all previous models. Results for miR-277 resembled those for the other two miRNAs (data not shown), even though miR-277 is unusual in that it primarily resides within Ago2 rather than Ago1 [2].

Using the mean fold change to evaluate repression (or de-repression) of top-ranked targets had several potential limitations. For example, it can exaggerate the influence of individual outliers or more heavily weight datasets with a greater variance in their fold-change distributions. Nonetheless, examination of plots showing the mean of median mRNA changes did not substantially change our assessment of the relative performance of each algorithm, which indicated that we did not arrive at erroneous conclusions because of outliers (Additional file 2: Figure S4). Another potential caveat is that our test sets looking at mRNA changes might miss targets that are repressed only at the level of translation, without changes in mRNA stability. Although such translation-only repression is widespread in early fish embryos [68, 69], examination of later embryos and post-embryonic mammalian cells and tissues has failed to find a set of targets convincingly regulated at only the level of translation [69–71], and we have no reason to suspect that such targets exist in the post-embryonic flies. Also potentially influencing our comparisons was the fact that for some previous algorithms predictions were missing for some miRNAs of our test sets. For example, EMBL predictions were not available for miR-263a and miR-994, and because targets for these two miRNAs happened to undergo less repression in our transfections, the testing of EMBL on only the remainder of the transfection datasets presumably inflated its relative performance.

Target-prediction algorithms have been developed with divergent priorities regarding prediction accuracy. Out of concern for prediction specificity, some, including our context model, consider only predictions with the most effective types of sites, i.e., 7–8-nt seed-matched sites within 3′ UTRs. In contrast, other algorithms, out of concern for prediction sensitivity, do not limit their predictions to those with these most effective site types, and some of these include predictions with a vast array of non-canonical sites that show no evidence of efficacy when tested using data from mammals and fish [51]. To begin to explore the tradeoffs of these divergent priorities when predicting miRNA targets in flies, we removed predictions containing 7–8-nt canonical sites to the cognate miRNA in their 3′ UTRs, and tested the behavior of the remaining predictions that lacked these more effective canonical sites. When testing on the transfection data, most algorithms that do not strictly focus on 3′ UTRs with 7–8-nt canonical sites generated predictions that were repressed more than expected by chance (Fig. 4c).

Encouraged by these results, we used our context features to build a model that considered predictions that lacked canonical 7–8-nt 3′ UTR sites but had at least one offset 6mer, 6mer, or 6mer-A1 site in their 3′ UTR. When using either test set and testing only predictions that lacked canonical 7–8-nt 3′ UTR sites to the cognate miRNA, this model, which we call the “6mer context” model, performed better than all existing algorithms, although statistically significant improvement was observed at only two thresholds when testing on de-repression of endogenous targets (Fig. 4c and d). The other algorithm that yielded predictions consistently repressed better than background was DIANA-microT-CDS, which includes predictions with only canonical ORF sites. Thus, taken together, our analysis indicates that two distinct strategies that focus on only marginally effective sites can be predictive in flies, as judged by both transfection and knockout results; one approach focuses on canonical 6-nt sites in 3′ UTRs, and the other focuses on canonical ORF sites. However, at best, the average repression of the four to eight top predictions from these approaches was much less than that of the top targets of the standard context model and instead resembled that of the hundreds of mRNAs that contained 7–8-nt canonical 3′ UTR sites (Fig. 4a–d).

The observation that models could be built that successfully predicted targets with only marginal canonical sites was consistent with the demonstrated efficacy of these marginal sites in Drosophila cells (Fig. 1). A larger challenge has been to predict effective non-canonical sites, which lack at least a 6-nt perfect match to the seed region. Although two types of non-canonical sites, known as the 3′ supplementary sites and centered sites, can mediate repression, these sites are rare—indeed so rare that is difficult to observe a signal for their action in mammalian cells without aggregating many datasets [5, 72]. Nonetheless, some algorithms yield many predictions that have only non-canonical sites. Analyses of mammalian datasets indicate that these predictions are no more repressed than expected by chance [51], raising the question as to whether any of the algorithms might successfully predict non-canonical sites in Drosophila. To answer this question, we used our two test sets to measure the response of predictions that lacked any canonical 6–8-nt site to the cognate miRNA in their 3′ UTR (Fig. 4e, f). The only predictions with a convincing signal above background in either test set were those of EMBL, DIANA-microT-CDS, and MinoTar. Manually examining the top-ranked predictions from EMBL revealed that the signal observed for its predictions was attributable to canonical sites located in ORFs and 3′ UTRs of alternative last exons, whereas the signal for the predictions of DIANA-microT-CDS and MinoTar was attributable to canonical ORF sites. We conclude that in flies, as in mammals [51], non-canonical sites only rarely mediate repression, although we cannot exclude the formal possibility that effective non-canonical sites are abundant yet for some reason not predicted above background by any of the existing algorithms.

### TargetScanFly (v7)

Having found that the context model performed better than the models that have been providing target predictions to the Drosophila research community (Fig. 4a, b), we overhauled TargetScanFly (available at targetscan.org) to display these improved predictions. Because of the diminishing returns of predicting targets with only marginal sites (Fig. 4c–f), we continued to limit TargetScanFly to predictions with 7–8-nt canonical 3′ UTR sites, with ranks driven by a version of the context model that was trained on the entire transfection dataset.

For simplicity, we had developed the context model using mRNAs without abundant alternative 3′ UTR isoforms (Fig. 3), and to make fair comparisons with the output of previous models, we had tested the context model using only the longest FlyBase-annotated isoform (Fig. 4). Nevertheless, because considering the usage of alternative 3′ UTR isoforms significantly improves the performance of miRNA targeting models [51, 60], our overhaul of the TargetScanFly predictions incorporated both the context scores and current isoform information when ranking mRNAs with canonical 7–8-nt miRNA sites in their 3′ UTRs.

Because the main gene-annotation databases (e.g., Ensembl/FlyBase) were still in the process of incorporating the information available on 3′ UTR isoforms, the first step in the overhaul was to compile a set of reference 3′ UTRs that represented the longest 3′ UTR isoforms for representative ORFs of the fly. These representative ORFs were chosen among the set of transcript annotations sharing the same stop codon, with alternative last exons generating multiple representative ORFs per gene. To compile this set of fly 3′ UTRs, we started with FlyBase annotations [73] for which 3′ UTRs were extended, when possible, using recently identified long 3′ UTR isoforms [74] and 3′-end reads marking additional distal cleavage and polyadenylation sites. The extension of these 3′ UTRs led to a substantial increase in the number of predicted regulatory interactions, with the median number of targets for conserved miRNAs increasing by 78% over the previous version of TargetScanFly (Additional file 2: Figure S5).

**Fig. 5:**
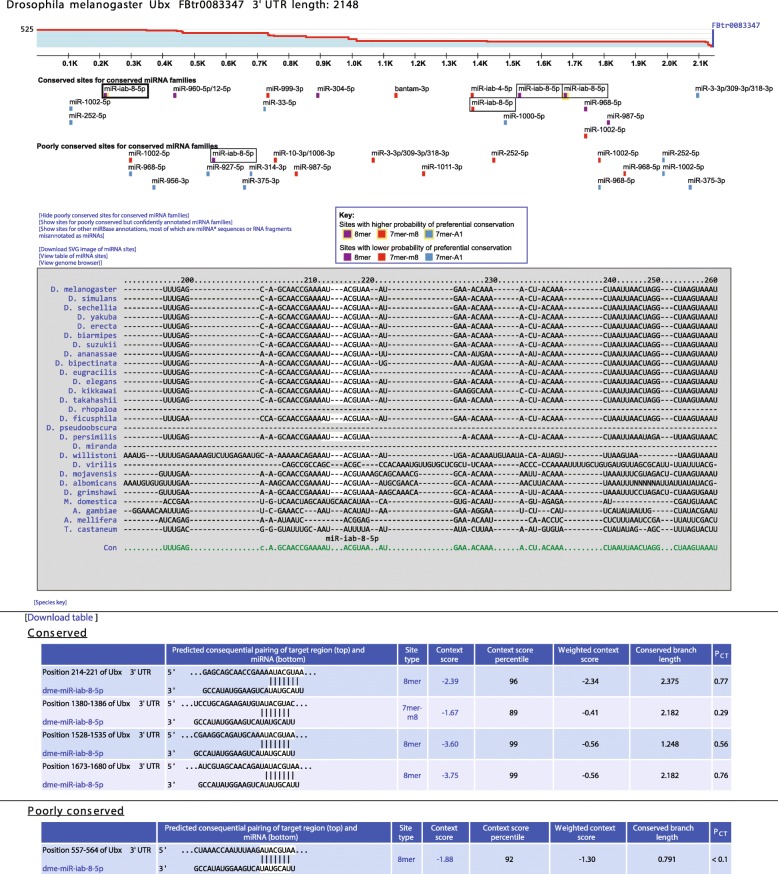
An example of a TargetScanFly page, which displays the predicted sites of conserved miRNAs within the *Ubx* 3′ UTR. At the top is the 3′ UTR profile, showing the relative expression of tandem 3′ UTR isoforms, as measured using 3′-seq [74] as well as our 3P-seq data. Shown on this profile is the end of the longest FlyBase annotation (*blue vertical line*) and the number of 3′-end reads (525) used to generate the profile (labeled on the *y*-axis). Below the profile are conserved and poorly conserved sites for miRNAs broadly conserved among insects (*colored according to the key*), with options to also display sites for poorly conserved miRNAs and other miRBase annotations. *Boxed* are the predicted miR-iab-8 sites, with the site selected by the user indicated with a *darker box*. The multiple sequence alignment shows the species in which an orthologous site can be detected (*white highlighting*) among 27 insect species. Below the alignment is the predicted consequential pairing between the selected miRNA and its conserved and poorly conserved sites, showing also for each site its position, site type, context score, context score percentile, weighted context score, branch-length score, and *P*_CT_ score

For each of these reference 3′ UTR isoforms, 3′-end datasets were used to quantify the relative abundance of tandem isoforms, thereby generating the isoform profiles needed to score features that vary with 3′ UTR length (len_3UTR and other_sites) and assign a weight to the context score of each site, which accounted for the fraction of 3′ UTR molecules containing the site [60]. Our 3P-seq data from S2 cells were combined with 3′-seq data from a range of developmental stages of the fly [74] to generate a meta 3′ UTR isoform profile for each representative ORF, as illustrated for *Ultrabithorax* (*Ubx*) (Fig. 5), which is known to undergo alternative cleavage and polyadenylation [75]. Although this meta approach is not expected to be as accurate as using individual datasets to generate isoform profiles and predictions tailored for an individual stage or cell type [61, 75–77], it simplifies the summary ranking of predicted targets for each miRNA and still outperforms the previous approach of not considering isoform abundance at all, presumably because isoform profiles for many genes are highly correlated in diverse cell types [60].

For each 7–8-nt canonical site, we used the corresponding 3′ UTR profile to compute the context score and to weight this score based on the relative abundance of tandem 3′ UTR isoforms that contained the site [60]. Scores for multiple sites to the same miRNA family were also combined to generate cumulative weighted context scores for the 3′ UTR profile of each representative ORF, which provided the default approach for ranking predicted targets with at least one 7–8-nt site to that miRNA family [51]. As an option, the user can instead request that predicted targets of broadly conserved miRNAs be ranked based on their aggregate *P*_CT_ scores [57], as updated in this study. The user can also obtain predictions from the perspective of each protein-coding gene, viewed either as the mapping of 7–8-nt sites shown beneath the 3′ UTR profile and above the 3′ UTR sequence alignment (Fig. 5), or as a table of miRNAs ranked by either cumulative weighted context score or aggregate *P*_CT_ score.

## Conclusions

At least 37% of Drosophila mRNAs are conserved miRNA targets. Thus, the scope of conserved targeting in flies is somewhat less than that of mammals but nonetheless a sizable fraction of the transcriptome. Although largely similar, miRNA targeting in flies and mammals has also diverged in important respects, which has led to more widespread efficacy of sites in Drosophila 5′ UTRs and altered features of site context that correlate with Drosophila site efficacy. A quantitative model of miRNA targeting in flies, which incorporates these insights and was uniquely developed and trained on high-throughput analysis of miRNA effects occurring in Drosophila cells, performs better than previous models. This model drives the latest version of TargetScanFly, which provides biologists with an interest in either a miRNA or a potential miRNA target convenient access to the predictions, with an option of downloading code or bulk output suitable for more global analyses. The accompanying TargetScanTools (https://github.com/vagarwal87/TargetScanTools) are also provided to help others reproduce and extend these analyses. These new insights and resources are anticipated to enhance the productivity of miRNA research in flies and thereby accelerate the understanding of this intriguing class of regulatory RNAs.

## Methods

### Cell culture

Drosophila Schneider 2 (S2) cells were grown in Express Five serum-free media (GIBCO) supplemented with glutamine to 16 mM. Upon reaching confluency (about every 3–5 days), cells were passaged following mechanical resuspension with a scraper (Corning). Prior to resuspension, the media and any unattached cells were removed and replaced with an equal volume of fresh media in order to select for attached cells.

### MicroRNA transfection, FACS, and mRNA isolation

Prior to transfection, cells were seeded into 6-well plates (Corning) at 2.5 × 10^6^ cells and 2 ml media per well. After 24 h, each well was co-transfected with 2.5 μg plasmid (25% p2032-GFP, 75% pUC19) plus 25 nM miRNA duplex (or for mock transfections, with plasmid only) using 5 μl DharmaFECT Duo (Dharmacon, Lafayette, CO, USA). Equal volumes of nucleic acid and DharmaFECT Duo diluted in 1× phosphate-buffered saline (PBS) were combined and incubated at room temperature for 20 min to form transfection complexes that were then added dropwise to the cells (500 μl/well). Twenty-four hours after transfection, cells were harvested, resuspended in 1× PBS, passed through a 70-μm filter, and stained with 5 μg/ml propidium iodide (PI). For each transfection, 3–5 × 10^6^ GFP-positive and PI-negative cells were isolated by FACS and lysed in 1 ml TRI Reagent (Ambion). Following extraction from the lysate, total RNA was cleaned up using the RNeasy Mini Kit (Qiagen, Hilden, Germany) and subjected to poly(A) selection using oligo(dT) Dynabeads (Invitrogen) to isolate mRNA.

### Preparation of sequencing libraries

Strand-specific mRNA-seq libraries for Illumina sequencing were prepared as described [71], with differences noted below. Briefly, poly(A)-selected RNA was hydrolyzed in alkaline buffer, resulting in fragments bearing 5′-hydroxyl and 3′-phosphate groups. Fragments between 36 and 55 nt were size selected, and end-specific adapters were sequentially ligated onto each terminus; prior to each ligation step, the appropriate 3′ or 5′ end chemistry was generated through dephosphorylation or phosphorylation, respectively. Adapter-flanked fragments were reverse transcribed and the resulting complementary DNA (cDNA) polymerase chain reaction (PCR)-amplified using primers complementary to each adapter. The PCR products were purified on a denaturing formamide gel and submitted for deep sequencing. 3P-seq libraries were prepared from RNA isolated from S2 cells as described [20].

### RNA-seq analysis

RNA-seq reads were analyzed using the quantification pipeline previously described [78, 79]. A genome index was built for the latest build of the *D. melanogaster* genome (dm6) using Spliced Transcripts Alignment to a Reference (STAR) v2.4 (options --runMode genomeGenerate --genomeFastaFiles dm6.fa --sjdbGTFfile dmel-all-r6.07.gff --sjdbOverhang 40 --sjdbGTFtagExonParentTranscript Parent) [80], with “dmel-all-r6.07.gff” referring to fly transcript models annotated in FlyBase release 6.07 [73], processed to have a single “Parent ID/exon” combination per line. Raw reads were aligned to the index with STAR (options --outFilterType BySJout --outFilterMultimapScoreRange 0 --readMatesLengthsIn Equal --outFilterIntronMotifs RemoveNoncanonicalUnannotated --clip3pAdapterSeq TCGTATGCCGTCTTCTGCTTG --outSAMstrandField intronMotif --outStd SAM). Considering all replicates of a particular sample, mRNA fold changes were computed between the miRNA transfection library of interest and the three mock-transfection biological replicates, using cuffdiff v2.2.1 (options --library-type fr-secondstrand -b dm6.fa -u --max-bundle-frags 100,000,000) [81], using protein-coding gene models from FlyBase release 6.07 [73].

### Selection of mRNAs for computational analysis

To avoid noisy mRNA fold-change measurements of poorly expressed genes, we used only genes whose expression values (measured in fragments per kilobase per million mapped reads, FPKM) exceeded 5.0 in the mock condition for all subsequent analyses. This threshold was chosen based upon visual inspection of plots evaluating the relationship between mean expression level and fold change (commonly known as “MA plots” in the context of microarrays), attempting to balance the tradeoff between sample size and noise reduction. To select gene annotations for site efficacy, data normalization, and evolutionary analyses (i.e., for Fig. 1, Additional file 2: Figure S1, and Fig. 2, respectively), we selected one representative transcript isoform per gene, choosing the transcript isoform with the longest ORF, and if tied, the one with the longest 3′ UTR, and if still tied, the one with the longest 5′ UTR. This representative transcript was supplemented with the longest 3′ UTR among the subset of transcripts that shared the same stop codon.

To select gene annotations for feature optimization and regression modeling (i.e., for Fig. 3 and Additional file 2: Figure S3A), we analyzed 3P-seq data to quantify the relative abundance of 3′ UTR isoforms related to each representative transcript. We then selected the subset of mRNAs for which ≥ 90% of the 3P-seq tags corresponded to a single dominant 3′ UTR isoform and used this dominant 3′ UTR isoform as the annotation for the corresponding gene. These steps followed the training framework previously described [51].

To select gene annotations for evaluation of model performance (i.e., for Fig. 4 and Additional file 2: Figure S4), we identified the longest and shortest 3′ UTR isoforms, as annotated by FlyBase, corresponding to each representative transcript. Context scores and aggregate *P*_CT_ scores were generated for the longest and shortest 3′ UTR isoform groups separately, and then, for each gene and miRNA combination, the scores were averaged between the longest and shortest isoforms. To filter out targets with a predicted target site (i.e., for Fig. 4b/d and Additional file 2: Figure S4), we removed those that contained the relevant site types in the 3′ UTR of their representative transcript.

### Dataset normalization

mRNA changes correlated among the six transfection experiments, indicating the presence of batch effects and other biases (Additional file 2: Figure S1A). To remove biases in the mRNA fold-change measurements, we implemented our previously described normalization strategy [51], which uses partial least-squares regression (PLSR) to remove sources of variation that are common to multiple independent miRNA transfections. This led to a modest improvement in our ability to detect signatures of miRNA-mediated target repression (Additional file 2: Figure S1B–D). However, 5′ UTR length, ORF length, 3′ UTR length, 5′ UTR AU content, ORF AU content, 3′ UTR AU content, and mock-transfection gene expression level still correlated with fold changes for mRNAs with no predicted miRNA target site. The magnitude of these correlations varied significantly when comparing the results of different miRNA transfection experiments. Thus, for each of the six miRNA transfection experiments, we fit a multiple linear regression model between the mRNA fold changes (i.e., which had already been normalized by the PLSR model) and the seven aforementioned features, using log-transformed values for the expression level feature. Although only mRNAs with no predicted canonical miRNA target site were used for this fit, the resulting linear model was used to predict mRNA fold changes for all mRNAs (including those with a predicted site), and for each gene, the residual value (the difference between the mRNA fold change and predicted mRNA fold change) was designated as its final normalized mRNA fold change (Additional file 1: Table S1). Applying this second normalization to data from each transfection experiment led to enhanced detection of target repression, as indicated by a shift towards more significant *P* values, especially for mRNAs with 3′ UTRs that contained weaker site types (Additional file 2: Figure S1D).

Each miRNA transfection exhibited a variable level of global target repression (Additional file 2: Figure S2). Reasons for this variability presumably included variability in transfection efficiency and differences in either the target abundance (TA) or the predicted seed pairing stability (SPS) of the miRNAs tested [51, 66]. Because we did not have the power in sample size to accurately model the effects of either SPS or TA, as was possible in mammals [51, 66], we normalized the transfections to the same scale prior to training and testing the model. To do so, for each transfection dataset *D*, we computed the upper and lower quartiles of the mRNA log fold changes (*UQ*_*D*_ and *LQ*_*D*_, respectively) as well as the corresponding quartiles for the fold changes among all datasets pooled together (*UQ*_*P*_ and *LQ*_*P*_). We then updated each fold change *x* as follows: $\widehat{x}=\left[\frac{x-{LQ}_D}{\left({UQ}_D-{LQ}_D\right)}\ \left({UQ}_P-{LQ}_P\right)+{LQ}_P\right]$. By centering on quartiles, this procedure normalized the fold-change distributions in a way that was less susceptible to the influence of outliers.

### Refining 3′ UTR isoform annotations

3P-seq data were processed as previously described [82] but with adjustment of some of the parameters to better fit the characteristics of the fly 3′ UTRs. Transcript models were identified using Cufflinks and the ModENCODE S2 RNA-seq data (Sequence Read Archive (SRA) accession SRR070279) [83] with default parameters and minimum intron length set to 10. 3P-seq reads were processed and aligned to the dm3 genome assembly as described [20], and the resulting tag positions were lifted over to the dm6 assembly using the University of California, Santa Cruz (UCSC) liftOver tool. In the first step of 3′ UTR annotation, clusters of 3P-seq tags were generated as described [82]. Briefly, positions were sorted in descending order based on read count, and the list was traversed such that, for the position with the highest read count (or the first encountered read, in the case of a tie), all the tags within 30 nt were grouped and removed from the list as a cluster. Each cluster represented by a position with at least three total reads and at least two unique reads was considered a poly(A) site and was assigned the representative position supported by the most reads. RNA-seq data were then used to test if the poly(A) site connected with transcript models, as described previously [82]. Connectivity to gene models was established based on the Cufflinks gene models, allowing for gaps of up to 200 nt. 3′ UTRs ending within 30 nt of each other were grouped together and assigned with their combined read count. The longest 3′ UTR of a gene was one with the maximal exonic length and which accounted for at least 1% of the 3P-seq reads. Other parameters were as described before [82]. A poly(A) site was considered to be “known” if it mapped within 20 nt of a FlyBase poly(A) site. 3p-seq tags mapped to the dm6 genome, processed into clusters, and annotated can be found as BED files associated with Fig. 3 at https://github.com/vagarwal87/TargetScanTools.

### MicroRNA sets

All mature fly miRNAs were downloaded from miRBase release 21 [15]. Those that matched a conserved miRNA at nucleotides 2–8 were considered part of that miRNA family. When partitioning miRNA families according to their conservation level, we compared the previously defined set of conserved families available in TargetScanFly v6 [8] with a more recent annotation of conserved “pan-Drosophilid” miRNA families [10]. For each difference between the two sets, we compared whether nucleotides 2–8 of each miRNA were conserved among most Drosopholids beyond the Sophophoran clade, as determined from the 27-way multiz alignments of each mature miRNA from the UCSC Genome Browser [84, 85]. This filter led to the removal of several miRNAs from being considered broadly conserved (e.g., mir-307b, mir-973, mir-975, mir-1014, mir-4977, and mir-4987) and the choice of a set of 91 conserved miRNA families (Additional file 4: Table S3). From these 91, the set of 28 families conserved since the ancestor of bilaterian animals was identified, starting with a previous annotation of bilaterian miRNA families [11], but separating related bilaterian families with different seed sequences and requiring that for each family the ancestral seed sequence has been conserved to Drosophila without a substitution or a shift in register (Additional file 4: Table S3).

A few conserved Drosophila primary microRNAs (pri-miRNAs) give rise to two abundant miRNA isoforms that have different seeds, either because both strands of the miRNA duplex load into Argonaute with near-equal efficiencies or because processing heterogeneity gives rise to alternative 5′ termini [8, 35]. To annotate these abundant isoforms, we identified all isoforms expressed with at least 33% of reads mapping to the same start position relative to the most abundantly mapped start position on the precursor hairpin, and if the sequences of these isoforms were conserved, the isoforms were included in the set of conserved miRNAs. Adhering to the miRNA naming convention, if two isoforms mapped to the 5′ and 3′ arms of the hairpin, they were named “–5p” and “–3p”, respectively, and if two isoforms were processed from the same arm, they were named “.1” and “.2” in decreasing order of their abundance, as detected in *D. melanogaster* (Additional file 4: Table S3). All miRNAs annotated in miRBase but not meeting our criteria for conservation were also grouped into families based on the identity of nucleotides 2–8 and were classified as either poorly conserved miRNAs or “other miRBase annotations” (which included many small RNAs misclassified as miRNAs). These miRNA seed families and their classifications are available for download at TargetScanFly (targetscan.org).

### Evolutionary analyses and calculation of *P*_CT_ scores

Fly *P*_CT_ scores were computed using the following datasets: (1) 5′ UTRs or 3′ UTRs, derived from 13,454 fly protein-coding genes annotated in FlyBase 6.07 [73], and (2) regions of multiple sequence alignments corresponding to these 5′ or 3′ UTRs, derived from the 27-way multiz alignments of the insect clade in the UCSC Genome Browser, which used the *D. melanogaster* genome release dm6 as its reference species [84, 85]. We partitioned 5′ UTRs and 3′ UTRs into five conservation bins based upon the median branch-length score (BLS) of the reference-species nucleotides, following the strategy previously described [20, 57]. BLSs were computed using the BranchLengthScoring.py script from MotifMap [86]. We used an updated computational pipeline for evolutionary analysis described previously [51] to estimate branch lengths of the phylogenetic trees for each bin, to compute the rates of *k*-mer conservation for canonical sites and control *k-*mers, and to calculate *P*_CT_ parameters and scores. All phylogenetic trees and *P*_CT_ parameters are available for download at our TargetScanTools GitHub page (https://github.com/vagarwal87/TargetScanTools).

### Estimating the number of genes with preferentially conserved sites

A simulation was performed to estimate the number of genes containing a conserved site after accounting for the background of conserved sites. Towards this goal, we first identified for each conserved miRNA all unique target sites with BLS ≥ 1.0, yielding a total of 8743 5′ UTR sites (considering 8mer, 7mer-m8, and 7mer-A1 sites) and 86,872 3′ UTR sites (considering 8mer, 7mer-m8, 7mer-A1, 6mer sites, and offset 6mer sites) that surpassed this cutoff. Among these, we estimated that 840 ± 40 5′ UTR sites and 12,285 ± 214 3′ UTR sites (mean ± standard deviation) were conserved above background. To estimate the distribution of genes with conserved sites, we performed 1000 samplings with the following procedure. (1) An integer was randomly selected from each of the two normal distributions of total sites above background. (2) Using each of these two integers, a corresponding number of conserved sites was randomly sampled (without replacement) from the respective 5′ UTRs or 3′ UTRs. (3) The number of unique genes containing the selected sites was recorded. After 1000 samplings, the distribution of values obtained for our estimate of genes with conserved sites had a mean of 5035 and a 90% confidence interval of ±83.

### Regression models

3P_energy was scored as described in the text. Other features were scored as described [51], except that SA was scored using the parameters optimized for Drosophila. For each feature of the final context model, scores were scaled (Additional file 2: Table S4) before being multiplied by their corresponding coefficients (Additional file 2: Table S5).

To evaluate performance, we generated 1000 bootstrap samples in which we used, for each site type and transfection experiment, 70% of data to train the models and the remaining data as a test set. To choose a model, we evaluated the performance of a variety of machine-learning strategies, including (1) “all subsets regression”, maximizing the Bayesian information criterion (BIC) as implemented in the *regsubsets* function of the “leaps” R package (parameters “nvmax=15, nbest=1, method=‘forward’, really.big=T”), (2) stepwise regression, maximizing the BIC or Akaike information criterion (AIC) as implemented in the *stepAIC* function from the “MASS” R package [87], (3) Lasso regression using the *cv.glmnet* function (parameters “nfolds = 10, alpha = 1”) in the “glmnet” R package, (4) multivariate adaptive regression splines (MARS) as implemented in the “earth” R package (parameters “degree = 1, trace = 0, nk = 500”), and (5) random forest regression using the “randomForest” R package, (6) principal component regression (PCR) or PLSR using the *pcr* and *plsr* functions as implemented in the “pls” R package (parameter “ncomp = 5” during prediction). As for our model of mammalian targeting [51], we ultimately utilized stepwise regression, with AIC to select features.

For the model driving TargetScanFly v7, we fit a multiple linear regression model for each site type using the selected group of features, training with all of the genes that were expressed above the threshold in our transfection datasets and had single 3′ UTR sites and 90% UTR homogeneity. As for mammalian predictions [51], scores for 8mer, 7mer-m8, and 7mer-A1 sites were bounded to be no greater than − 0.03, − 0.02, and − 0.01, respectively, thereby creating a piecewise linear function for each site type. For each 3′ UTR with at least one 7–8-nt site to the miRNA, the context scores of the sites were weighted based on the UTR profile, and multiple weighted scores for the same miRNA were combined to generate a cumulative weighted context score, which was used to rank the predicted target gene.

### Performance comparisons

To compare predictions from different miRNA target-prediction tools, we collected the following downloadable predictions: ComiR (October 2015) [49], DIANA-microT-CDS (September 2013) [46], EIMMo v5 (January 2011) [41], EMBL (2005 predictions) [6, 40], miRanda-MicroCosm v5 [42], mirSVR (August 2010) [47], PicTar (from the doRina web resource; sets conserved among *D. melanogaster*, *D. yakuba*, *D. ananassae*, *D. pseudoobscura*, *D. mojavensis*, and *D. virilis*) [16, 43], PITA Catalog v6 (3/15 flank for either “All” or “Top” predictions, August 2008) [38], RNA22 (May 2011) [44], RNAhybrid [45], TargetSpy (all predictions) [48], MinoTar (downloaded from TargetScanFly ORF v6.2, June 2012) [19], and TargetScanFly v6.2 (June 2012) [8]. For algorithms providing site-level predictions (i.e., ElMMo, mirSVR, PITA, and RNA22), scores were summed within genes or transcripts (if available) to calculate an aggregate score. For algorithms providing multiple transcript-level predictions (i.e., DIANA-microT-CDS, miRanda-MicroCosm, and TargetSpy), the transcript with the best score was selected as the representative transcript isoform. In all cases, predictions with gene symbol or RefSeq ID formats were translated into FlyBase format. To avoid testing and training our context model on the same data, we generated cross-validated predictions for the context model. To do so, we held out each transfection dataset, fit a linear regression model using the data from the remaining five datasets, and generated predictions on the held-out data.

### Microarray processing

We downloaded raw Affymetrix data measuring the effects of a miR-14 knockout (GEO accession GSE20202) [31], a miR-34 knockout (day 20, GEO accession “GSE25008”) [32], and a miR-277 knockout (ArrayExpress accession “E-MEXP-3785”) [33] and processed the data as previously described [51], with the exception that the *drosophila2FLYBASE* function in the “drosophila2.db” R Bioconductor package was used to map Affymetrix probe IDs to FlyBase IDs.

### 3′ UTR profiles for TargetScanFly (v7) predictions

In addition to our 3P-seq data from S2 cells, we downloaded *D. melanogaster* 3′-seq data for the following tissues and cells: carcass female, carcass male, embryo 0–45 min, embryo 1.5–6 h, embryo 6–12 h, embryo 12–18 h, embryo 18–24 h, female head, ovary, S2R+, testis, whole body male 2–5 days (d), and whole body female 2–5 d [74]. The dataset for embryo 45–90 min was excluded due to poor library quality. To process the 3P-seq and 3′-seq reads, Illumina adapters were trimmed from all sequences using Trimmomatic. All terminal adenosines were then trimmed from the remaining sequence, and the subset of reads that were at least 20 nt long after trimming and had possessed at least two terminal adenosines was carried forward. These reads were mapped to the dm6 genome and processed as previously described [20].

To build fly 3′ UTR profiles, we began with the set of protein-coding gene models deposited in FlyBase 6.19 [73]. For each unique stop codon in each set of gene models, we selected the transcript with the longest 3′ UTR as the transcript with the reference 3′ UTR. For the 3′ UTR associated with the most distal stop codon, we extended it if a longer tandem isoform was supported by RNA-seq and 3′-seq evidence (Additional File 7 of Sanfilippo et al. [74]). For any 3′ UTR associated with a stop codon whose exon overlapped the exon harboring the most distal stop codon, we extended the 3′ UTR to the end of the longest 3′ UTR isoform associated with the most distal stop codon. Finally, for a 3′ UTR associated with a stop codon located in an upstream alternative last exon, we used 3′-end tags to further extend 3′ UTRs when possible, searching within the intronic region downstream of the stop codon for a cleavage and polyadenylation site supported by at least ten 3′-end reads (pooling read counts across all samples), prohibiting the search to extend beyond the start position of any annotated downstream exon. For each reference 3′ UTR, 3′-end reads from both 3P-seq and 3′-seq were normalized for sequencing depth across cell lines/tissues and used to quantify the relative levels of alternative tandem isoforms, thereby generating a 3′ UTR profile [51].

### TargetScanFly predictions

TargetScanFly (v7) provides the option of ranking predicted targets of mammalian miRNAs according to either cumulative weighted context score, which ranks based upon the predicted repression, or aggregate *P*_CT_ score of the longest 3′ UTR isoform, which ranks based upon the confidence that targeting is evolutionarily conserved. For each predicted target, the cumulative weighted context score estimated the total repression expected from all of the sites to the same miRNA family. This score was calculated starting with the context score of each site to a miRNA family, calculated using the model trained on all of the transfection data (Additional file 2: Table S5) and using the 3′ UTR profile to weight the predicted effect of the most proximal site and the marginal effects of any additional sites [51]. When scoring features that can vary with 3′ UTR length (Len_3UTR and Other_sites), a weighted score was used that accounted for the abundance of each 3′ UTR tandem isoform in which the site existed, as estimated from the 3′ UTR profile. When calculating *P*_CT_ scores, if alternative 3′ UTRs were annotated for the same gene, the most conserved 3′ UTR isoform was used to determine the conservation bin to which the 3′ UTR belonged. Sites corresponding to poorly conserved miRNA seed families or sites overlapping annotated ORF regions were assigned *P*_CT_ scores of zero.

Predictions were generated for the transcript associated with each UTR profile. For genes with multiple UTR profiles, each associated with an alternative transcript with a unique stop codon, we chose a single transcript to represent that gene in the default predictions and target rankings. This representative transcript was chosen as the alternative with the longest ORF that had at least 60% of the maximum number of normalize 3′-end tags for any transcript of that gene, unless its UTR overlapped another UTR of the same gene, in which case, we chose the transcript with the longest UTR. Users interested in predictions for alternative transcripts not chosen as the representative transcript can access those predictions by starting a search based on their gene of interest.

All predictions for representative transcripts and input and output annotation files as well as associated scripts are available for download at TargetScanFly (targetscan.org) or our TargetScanTools Github page (https://github.com/vagarwal87/TargetScanTools). All *P*_CT_ parameters and parameters for tree branch lengths and regression models, along with pre-computed context scores, are also available (targetscan.org).

## Additional files

Additional file 1: **Table S1.** Processed mRNA abundances (measured in fragments per kilobase per million mapped reads (FPKM)) and mRNA fold changes corresponding to each of the six miRNA transfection datasets. (XLSX 4739 kb)

Additional file 2: Supplementary figures, Table S4, and Table S5. (PDF 3961 kb)

Additional file 3: **Table S2.** *P* values reporting the significance of the differences in fold-change distributions observed between site types for each of the three mRNA regions (3′ UTR, ORF, and 5′ UTR). (XLSX 12 kb)

Additional file 4: **Table S3.** The 91 seed families broadly conserved in Drosophila species, listing for each family the miRNA names, seed sequence, and signal-to-background ratios for 5′ UTR and 3′ UTR sites. These ratios are plotted in Fig. 2f. Families conserved since the ancestor of bilaterian animals are also indicated. (XLSX 14 kb)
