# Supplementary material for: Predicting microRNA targeting efficacy in Drosophila
Source: Genome Biol. 2018 Oct 4;19:152. doi: 10.1186/s13059-018-1504-3 (PMC6172730; doi:10.1186/s13059-018-1504-3)
Supplement: Supplementary file 2 — Supplementary figures, Table S4, and Table S5. (PDF 3961 kb) [file 13059_2018_1504_MOESM2_ESM.pdf]

SUPPLEMENTAL TABLES

Table S4. Scaling parameters used to normalize data to the [0, 1] interval. Provided are the 5th and 95th percentile values for continuous features that were scaled, after the values of the feature were determined and transformed as indicated (Table 1).

	8mer		7mer-m8		7mer-A1	
**Feature**	**5th %**	**95th %**	**5th %**	**95th %**	**5th %**	**95th %**
3p_energy	−4.740	0.000	−3.950	0.000	−3.935	0.000
Other_sites	0.000	1.400	0.000	2.750	0.000	2.000
Len_3UTR	1.957	3.190	1.960	3.144	1.962	3.165
Len_ORF	2.671	3.632	2.620	3.661	2.639	3.712
SA	−4.933	−0.791	−5.767	−0.800	−5.464	−0.939
P_{CT}	0.000	0.891	0.000	0.825	0.000	0.760

Table S5. Coefficients of the trained context model corresponding to each site type, as shown in Figure 3E. Using the corresponding scaling factors (Table S4) and these coefficients, context scores of predicted targets can be computed as described [1].

	8mer	7mer-m8	7mer-A1
(Intercept)	−1.593	−1.066	−0.632
3p_energy	0.237	0.309	0.174
Other_sites	−0.368	−0.378	−0.118
Len_3UTR	0.991	0.940	0.498
Len_ORF	0.822	0.294	0.178
SA	−0.375	−0.338	−0.237
P_{CT}	−0.391	−0.357	−0.376

Figure S1

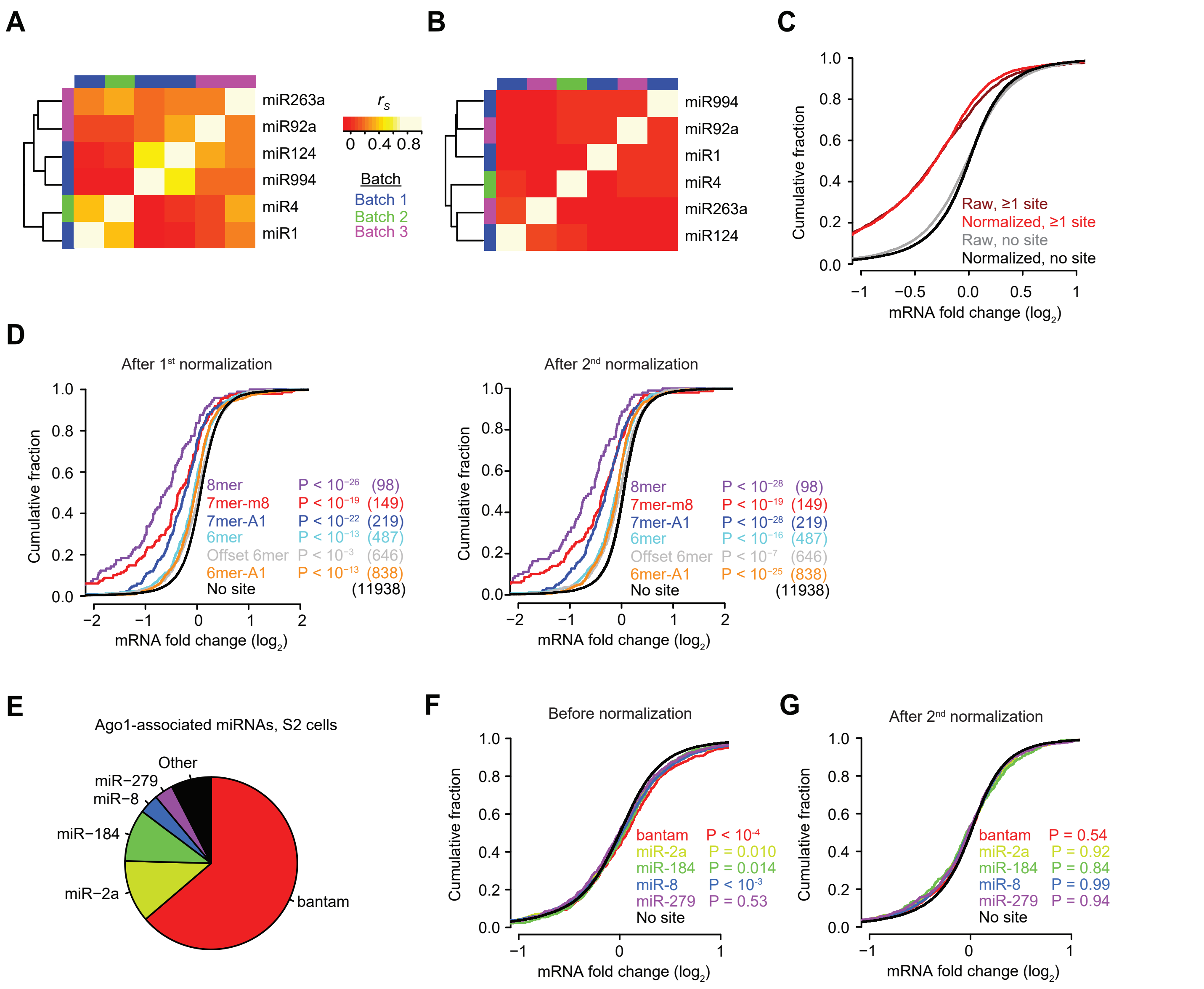

Figure S1. Preprocessing of RNA-seq datasets to minimize non-specific effects and technical biases.

(A) Correlations observed between the responses of mRNAs without canonical 7–8-nt 3′-UTR sites to the transfected miRNAs. For each pair of experiments, the Spearman correlation (r_s) of fold-change values for these mRNAs was calculated, and these r_s values, colored as indicated in the key, were then used for hierarchical clustering. The six transfection experiments were performed in three separate batches, which are colored as indicted in the batch list to show the correspondence between the clustering and the batches. **(B)** Reduced correlations observed between the responses of mRNAs without canonical 7–8-nt 3′-UTR sites to the transfected miRNAs after applying the PLSR technique. This heat map is as in (A) but plots the r_s values obtained using PLSR-normalized mRNA fold changes. **(C)** Effects of the PLSR-based normalization on the fold-change distributions. Plotted are cumulative distributions of fold-changes observed after transfection of each of the six miRNAs, showing results for mRNAs containing either no site or at least one canonical 7–8-nt 3′-UTR site, either before (raw) or after PLSR-based normalization (normalized). **(D)** Residual mRNA fold changes either before (left) or after (right) a second round of normalization that removed biases between the mRNA fold changes and the A/U composition and sequence length of 5′ UTRs, ORFs, and 3′ UTRs. The panel showing results after the second round of normalization is the same as Figure 1C. **(E)** The most abundant miRNAs within the Ago1 of S2 cells. The pie chart reflects the relative proportions of reads for the indicated miRNA families observed when sequencing Ago1-associated small RNAs from S2 cells [2]. **(F and G)** The effect of normalization on the observed de-repression of endogenous miRNA targets. Plotted are cumulative distributions of fold changes for mRNAs with at least one canonical 7–8-nt 3′-UTR site to the indicated miRNA family in the compendium of 6 miRNA transfection datasets, either before any normalization (F) or after the second normalization (G). *P* values were computed using a one-sided Wilcoxon rank-sum test, comparing each of the site-containing distributions to the no-site distribution. This test was a more stringent alternative to the K–S test, which led to highly significant *P* values for very slight differences, due to the large number of mRNAs in each distribution.

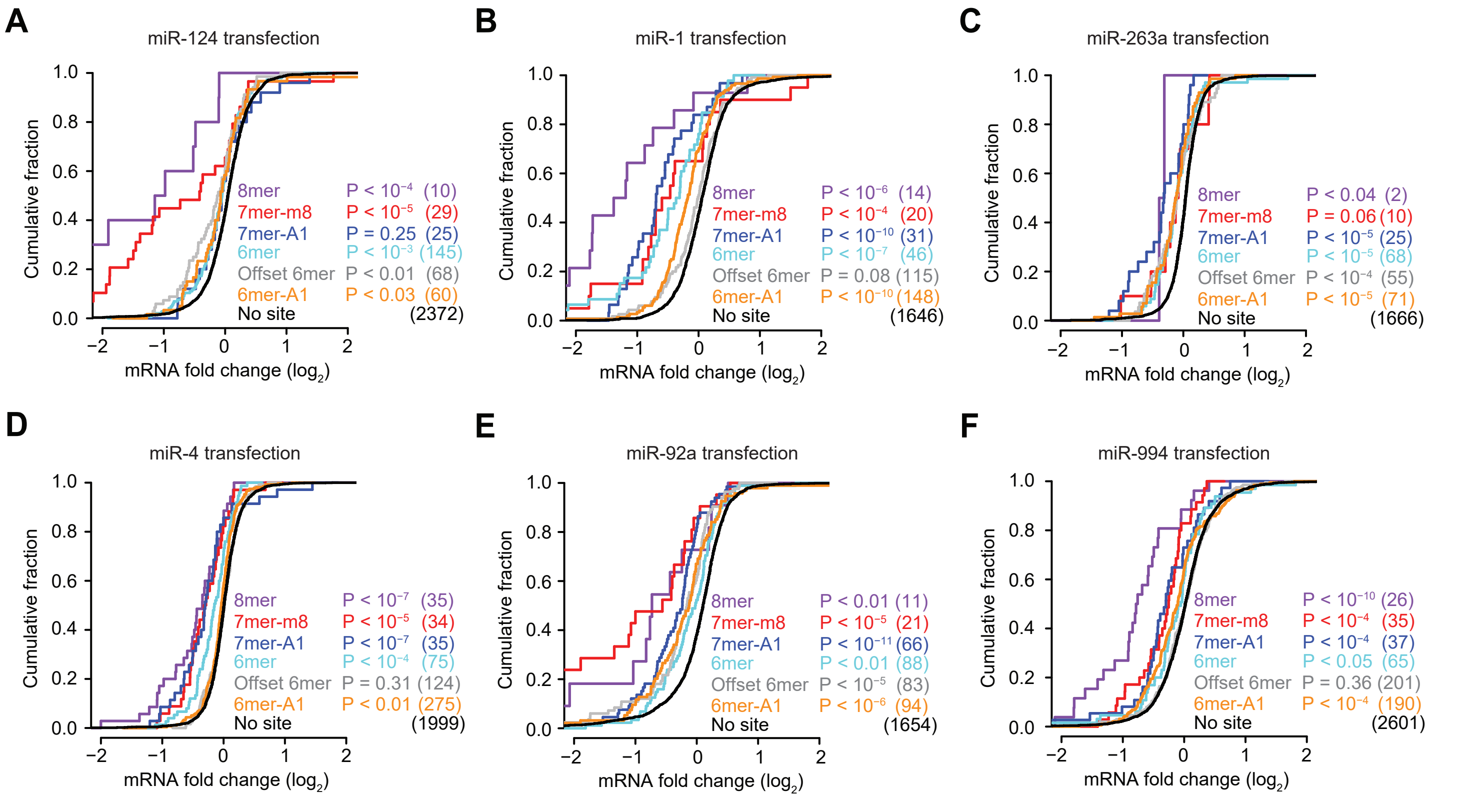

Figure S2. The efficacy of the canonical site types observed in Drosophila 3′ UTRs for individual experiments transfecting miR-124 **(A)**, miR-1 **(B)**, miR-263a **(C)**, miR-4 **(D)**, miR-92a **(E)**, or miR-994 **(F)**. Otherwise, these panels are as in Figure 1C.

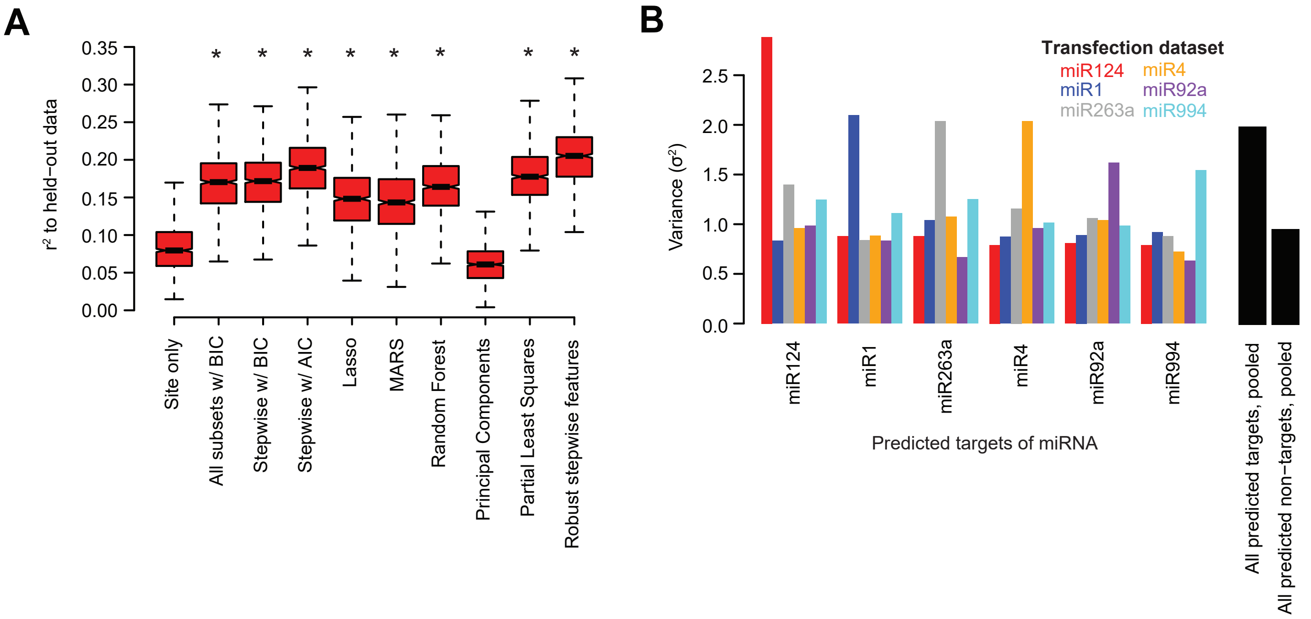

Figure S3. Evaluation of different machine learning algorithms.

(A) Performance of the models generated using different stepwise-regression methods compared to that of the site-only model. Shown are boxplots of r^2 values for each of the models across all 1000 sampled test sets. Highly significant improvement from the site-only model is indicated (*$P < 10^{-15}$, paired Wilcoxon sign-rank test). Boxes indicate the median and interquartile ranges, and whiskers indicate either 1.5 times the interquartile range or the most extreme data point. **(B)** Estimate of the variance attributable to direct miRNA targeting. To estimate this variance, we examined the variance of the predicted targets of a miRNA observed when the cognate miRNA was transfected and compared it to the variance of these predicted targets observed when other miRNAs were transfected. mRNA fold changes for each transfection dataset were z-score normalized to standardize each of the datasets to a comparable scale. Predicted targets of each miRNA (i.e., those possessing at least one 3′-UTR 7mer-A1, 7mer-m8, or 8mer site) were identified, and the variance for their corresponding fold changes in each of the transfections was computed, after removing fold-changes for mRNAs that were predicted co-targets for the transfected miRNA. These variance values are plotted using narrow bars, colored according to the key. The variance of the fold changes of the predicted targets in the cognate transfection datasets was 1.967, whereas the variance of the fold changes of the predicted targets of miRNAs transfected in other datasets was 0.949 (thick black bars). Because 1.967 represents the variance of the primary and secondary effects of a miRNA transfection as well as measurement noise ($\sigma^2_{primary} + \sigma^2_{secondary} + \sigma^2_{noise}$) and 0.949 represents the variance attributable to secondary effects and measurement noise ($\sigma^2_{secondary} + \sigma^2_{noise}$), the variance in panel (A) attributable to primary effects is (1 – 0.949/1.967), or an r^2 of 0.517.

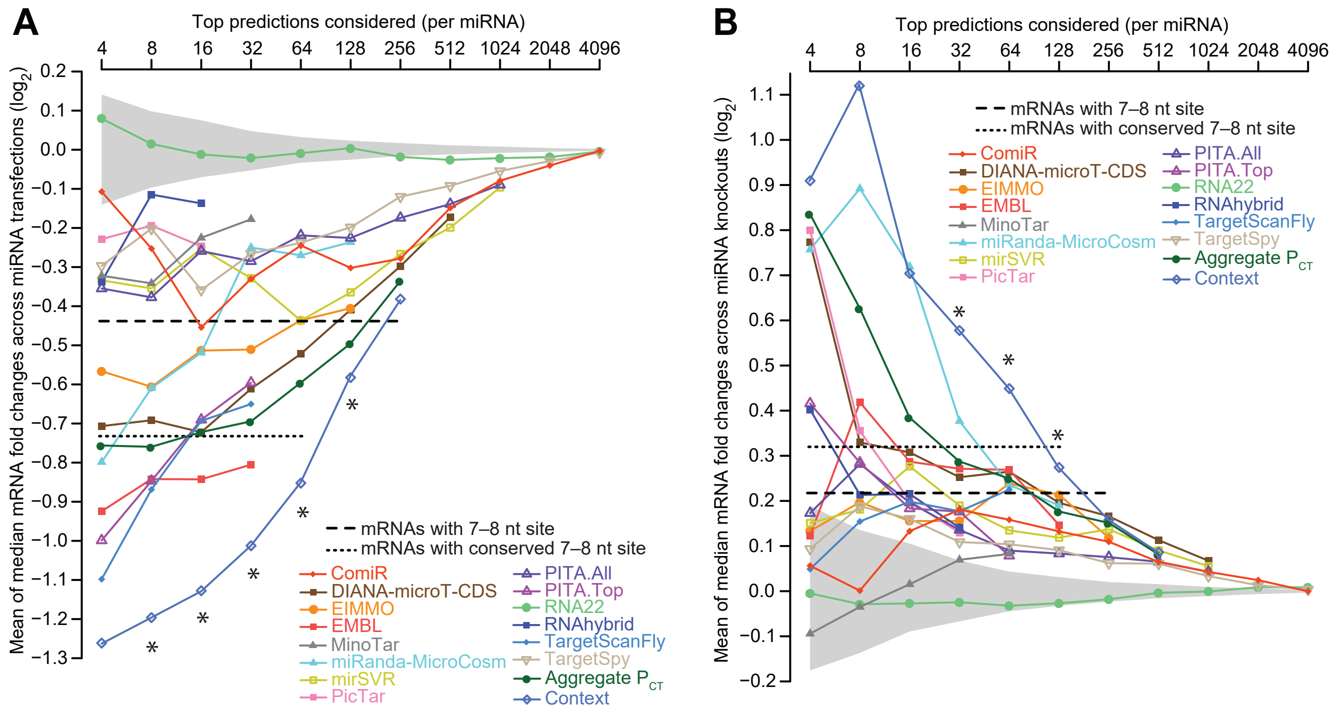

Figure S4. An alternative analysis of target-prediction performances in flies.

(A and B) Evaluation of prediction performance plotting the mean of median values instead of the mean of mean values. Otherwise these panels are as in Figure 4A and B, respectively.

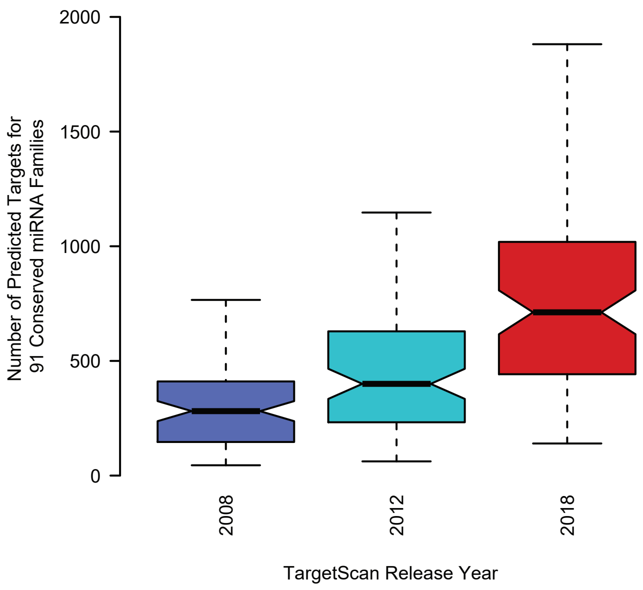

Figure S5. The increase in predicted targets with subsequent releases of TargetScanFly.

Plotted is the distribution of predicted targets for the conserved miRNA families in the indicated releases (boxplots as in Figure S3A). For releases in the years 2008, 2012, and 2018, the median number of predicted targets per conserved miRNA families was 281, 400, and 712, respectively. The 78% increase observed between the current release (TargetScanFly v7) and previous release (TargetScanFly v6.2) was largely attributable to improved 3′ UTR annotations.

SUPPLEMENTAL REFERENCES

1. Agarwal V, Bell GW, Nam JW, Bartel DP. Predicting effective microRNA target sites in mammalian mRNAs. Elife 2015, 4.
2. Czech B, Malone CD, Zhou R, Stark A, Schlingeheyde C, Dus M, et al. An endogenous small interfering RNA pathway in Drosophila. Nature 2008, 453:798-802.
